# A Study of Older Adults’ Perception of High-Density Housing Neighbourhoods in Singapore: Multi-Sensory Perspective

**DOI:** 10.3390/ijerph18136880

**Published:** 2021-06-26

**Authors:** Zdravko Trivic

**Affiliations:** Department of Architecture, School of Design and Environment, National University of Singapore, Singapore 117566, Singapore; akizt@nus.edu.sg

**Keywords:** age-friendly neighbourhood, multi-sensory experience, perception, ageing population, high-density environment

## Abstract

Associated sensory and cognitive declines progress with ageing and profoundly impact the daily living and quality of life of older adults. In the context of an increased ageing population globally, this paper outlines an exploratory study of socio-sensory properties of two high-density housing neighbourhoods in Singapore and the ways senior local residents perceive their familiar built environments. This study employed exploratory on-site exercises with 44 student researchers (including sensory photo-journeys, documentation of sensory properties and daily activity patterns), and 301 socio-perceptual surveys with local residents, the majority of whom were older adults. The findings reveal important aspects related to sensory assessment and appreciation (e.g., crowdedness, noise, smell, cleanliness), walking experience (e.g., safety, wayfinding) and overall satisfaction with the neighbourhood (e.g., available public amenities, opportunities for inter-generational bonding), some of which correlated with age and reported health condition. Multi-sensory assessment shows the capacity to inform more integrated, empathetic, ability-building and context-specific ageing-friendly neighbourhood design.

## 1. Introduction

The ways people experience, understand and use built environments are shaped by the dynamic and interdependent bodily, emotional, cognitive and symbolic everyday interactions with spaces (e.g., [1,2]). According to humanistic geographer Yi-Fu Tuan [3], multi-sensory apprehension is the most profound mode of experiencing a place, whereby place reaches concrete reality only when people’s experience of it is total, through actively and simultaneously engaged senses and mind. Multi-sensory experience substantially affects individuals’ overall sense of physical, psychological and social well-being (see, e.g., [4,5]). However, the predominant design preoccupation with sight (over other senses) has led to the production of either sensory-overloaded or sensory-deprived built environments, resulting in substantial erosion of our perceptual sphere (see, e.g., [2,6,7]). The decline in sensory (and cognitive) capacity is one of the common consequences of ageing, yet underrated and empirically understudied (e.g., [8]), especially in the context of age-friendly neighbourhood design. With an increasing ageing population globally, it is important to explore how seniors perceive their living environment in order to better inform the design of healthful, ability-building, empathetic and inclusive environments for all ages.

### 1.1. Ageing Population Trends

The latest World Population Prospects 2019 predicted the growth of the global population over age 65 to increase from 9.1% in 2019 to 11.7% in 2030, and to 15.9% by 2050 [9]. According to the same source, in East and Southeast Asia, the population aged 65 or over will increase from 11.2% in 2019 to 23.7% by 2050. Such estimates are even more dramatic in Singapore, where the population aged 65 or above is anticipated to rise from the current 716,000 (12.4%) to 1,409,000 (22.5%) by 2030, and to 2,132,000 (33.3%) by 2050 [10]. Moreover, the number of older Singaporeans (aged 65 and above) living alone is estimated to increase from 67,600 in 2019 [11] to 83,000 by 2030 [12].

### 1.2. Sensory Decline

A study by Correia and colleagues [13] showed that 94% of people aged 57 or above have at least one sensory impairment. The most common of such impairments are declines in motor abilities, and weakening of visual and auditory functions and odour detection. Estimations state that at least 2.2 billion people globally (or around 28%) have some degree of visual impairment, with ageing being the main risk factor [14]. Among the total number of visually impaired people worldwide, people aged 50 or above comprise more than 65% [15]. A study of 3353 Singaporeans aged 40 and above reported 21.4% of participants with bilateral visual impairment or blindness, the vast majority of whom were aged 60 and above [16]. Poor vision is associated with limitations in physical functioning, a higher risk of falls and injury, lower performance in basic daily activities (e.g., [17]), dissatisfaction with social life and poor self-reported health (e.g., [18]) and, finally, higher mortality (e.g., [19]).

Around 30–60% of adults aged 65 and above and 70–90% above the age of 85 report some degree of hearing impairment [20]. A study conducted in Singapore estimated that in 2017, 63.7% of Singaporean elderly (aged 60 and above) had some degree of hearing impairment and 16.2% had disabling hearing loss [21]. People with various types of hearing impairment are more likely to have communication difficulties and poorer self-esteem, which often leads to withdrawal from social interaction, depression, loneliness and various other psycho-social problems [22,23].

About 24% of people aged above 70% and 60% of those aged above 80 show problems with odour identification [24,25]. Similarly, over 60% of adults older than 70 show taste deficits [26]. Tactile impairment also prevails among adults aged over 55 [27]. Finally, almost 50% of adults aged over 80 have some degree of motor impairment [28]. In Singapore, it has been reported that 17.2% of older adults experience at least one fall per year [29].

### 1.3. Cognitive Decline

In 2019, the estimated number of persons with dementia globally was about 50 million, with predictions to rise to 82 million by 2030 and to 150 million by 2050, whereby 5–8% of adults older than 60 will have dementia [30]. A study by Subramaniam and colleagues [31] reported that in 2015, 10% of Singaporeans aged 60 years and above had dementia, which translates to about 82,000 people in 2018, and over 100,000 by 2030.

Numerous studies indicated that sensory declines increase the chance of developing various cognitive impairments and psychological conditions, including dementia (e.g., [32]), delirium (e.g., [33]) and depression (e.g., [34]), among others. A study by Feng and colleagues [35] indicated that physically frail older Singaporeans (aged 55 and above) are more likely to have cognitive impairment, whereby almost 40% of frail elderly were cognitively impaired. Moreover, the elderly with dual sensory impairment, which typically refers to concurrent hearing and visual declines, are at a higher risk of cognitive impairment, depression and social and communicational problems than those with single sensory impairment (e.g., [36]).

Finally, studies show that both sensory and cognitive impairments negatively affect numerous aspects of senior adults’ everyday life (e.g., [37,38]), such as a reduced level of mobility and physical activity, impoverished navigation and spatial orientation abilities and communication difficulties, among others. This often further results in decreased levels of confidence and autonomy, which is reflected in lower levels of participation in outdoor and social activities (e.g., [39,40]).

### 1.4. Sensory Approaches in Architecture and Urban Design

While addressing multi-sensory experience has historically been an integral part of architectural and urban design discourses and practices (see, e.g., [2,41]), empirical studies of human perception in respect to health- and ageing-supportive neighbourhood design are relatively scarce. Some of the reasons may be due to numerous challenges to identify, document, measure, evaluate and represent multi-sensory experiences, which are subjective and difficult to articulate.

Contrary to the 19th century, when the major health issues in cities were caused by poor hygiene and overcrowding conditions, the vast majority of health problems in contemporary cities arise from an inactive lifestyle and stress (e.g., [42]). Numerous concepts and studies, predominantly stemming from healthcare discourses, such as ‘attention restoration theory’ [43], ‘theory of supportive healthcare design’ [44], ‘total healing environment’ [45] and the concept of ‘salutogenesis’ [46], among others, have demonstrated an active role of built and natural environments in triggering, restoring and maintaining health outcomes. Well-designed and aesthetically pleasant spaces and nature contribute to holding attention, distracting from worrisome and stressful thoughts, prompting positive emotions, boosting self-esteem and improving physical, cognitive and emotional functioning and, therefore, to the people’s overall sense of well-being.

In the context of healthcare settings, since the 1970s, multi-sensory environments (MSE) have been used to treat various sensory disorders, cognitive impairments, depression and anxiety, and behavioural and learning difficulties, among others, through sensory stimulation (e.g., [47,48]). Drawing from the ‘ecological model of ageing’ [49], and the concepts of ‘person–environment fit’ (e.g., [50]) and ‘enabling environment’ (e.g., [51]), some studies of nursing home outdoor environments (especially gardens) explored sensory stimulation strategies not only to compensate for the loss of sensory and mental capabilities but also to restore and build such abilities (e.g., [52,53,54]). A study by Bengtsson and Carlsson [55] proposed a combination of compensatory and advancement stimulation to offset functional and cognitive declines (precautionary design) and to challenge abilities through motivation and intrigue (inspiring design). In another study, sensory-based interventions were proposed to shift the focus from seniors’ disabilities and instead tap on and empower the use of the remaining abilities [56]. However, the capacity of neighbourhood design to embed carefully orchestrated multi-sensory stimuli and, in such a way, become an integral and active component of care and ability building has been largely neglected.

The turn of the 21st century witnessed a growing interest in embodied experience and sensory studies (e.g., [57,58]), which has been described as the ‘sensory revolution’ [59] or ‘sensory turn’ [60,61] in humanities and social sciences. Recent advances in neuroscience, cognitive science and post-phenomenology also brought new perspectives on multi-sensory and embodied spatial experience in architectural and urban design discourses, particularly in the form of synaesthetic and human-centred design (see, e.g., [62,63,64]), what Jelić and colleagues [65] call an ‘experiential turn’.

Such renewed interest builds upon the pioneering works in phenomenology, anthropology and humanistic geography, which represented a critical response to the hegemony of vision (and language), Cartesian duality between body and mind, and under-representation and under-investigation of other senses and overall embodied experience in research. Husserl’s ‘intentionality’ [66], Heidegger’s ‘being-in-the-world’ [67] and Merleau-Ponty’s ‘phenomenal field’ [1] are some of the main phenomenological concepts that have substantially shaped the contemporary work in sensory studies. Of particular importance for architectural phenomenology is Christian Norberg-Schulz’s focus on phenomenology of urban spaces and the concept of *genius loci* [68,69], which differs from more prevailing writings concerned with the phenomenology of home and the sense of ‘being at home’ (see, e.g., [70,71]). For Dovey [72], ‘being at home’ does not refer to being in an interior space, being sheltered or dwelling in one place, or to a nostalgic return to past values, alluding to Heidegger’s notion of ‘being in the world.’ It rather refers to ontological security, a strong sense of cohesion or emotional connection between people and built form, which is fundamental to human health and well-being. ‘Home’ also does not refer to a fixed space, but rather a series of routine and often mundane practices, which contribute to the creation of place identity and the production of self [73,74]. Understanding the ‘sense of place’ and the subjectivity of place experience was at the core of research of human geographers in the 1970s (see, e.g., [3,75,76,77,78]). According to Yi-Fu Tuan’s [78] ‘topophilia’ (‘love of place’), places create ‘fields of care’, which depend on the emotional investment that people make in different places. Moreover, Doreen Massey [79] highlighted space–time integration and proposed a more dynamic interpretation of ‘sense of place’, which is defined by the multiple identities and the Deluzean ‘becoming in the world’ [80,81], rather than the Heideggerean ‘being in the world’ [67]. In architecture and urban design disciplines, several recent studies focused on approaches to capturing, measuring, evaluating, analysing and visualising sensory qualities of urban environments and subjective multi-sensory experiences (e.g., [82,83,84]). Among these, a study by Lucas and Romice [85] represents one of the rare attempts to systematically employ sensory experience in urban design research and practice, based on notational systems and sensory multi-modality. Their sensory notation system builds mainly upon the phenomenology of Maurice Merleau-Ponty [1], which focuses on perception rather than on being and experience (discussed by Heidegger), and on description rather than analysis. Moreover, the authors build upon James Gibson’s [86] perceptual systems and sensory classification while also shifting the focus from the ‘space’ as a neutral and constant construct to the ‘medium’, which is fluid, changeable and contextual (see also [87]). Similarly, yet from a sensory ethnography perspective, Palipane [88,89] proposed a framework of sensory production of urban space based on socio-sensory perception and multi-modal mapping techniques for documenting diverse multi-cultural place-making practices. Several other studies (see, e.g., [90,91,92,93]) also link senses and ‘sensescapes’ to the quality of urban spaces and place-making practice.

### 1.5. Research Objectives

The overarching aim of this research is to investigate how residents, and particularly senior adults, perceive and appreciate their outdoor living environments from the perspective of multi-sensory experience. The main premise is that a better understanding of socio-sensory properties of built environments can contribute to better design and planning of healthful and age-friendly neighbourhoods that would enable more meaningful and joyful ‘ageing in place’ and ‘active ageing’ for all.

This study employs an exploratory multi-sensory approach to audit socio-sensory properties of two high-density neighbourhoods in Singapore. Upon a brief review of the relevant literature, Section 2 presents research methods, considering two phases of research. The first phase involved on-site socio-sensory exercises with student researchers, with an aim of exploring various methodologies and techniques and gaining the initial insights about the neighbourhoods’ socio-sensory properties and rhythms. In this article, such insights are presented primarily to provide the necessary context of the two study areas and to inform the subsequent phase of the study. The second phase, which is the main focus of this article, comprised socio-perceptual surveys to investigate how senior local residents perceive their familiar built environments, and to uncover potential correlations between socio-sensory perception, age and self-reported health condition. Section 3 presents the key findings from both phases of the study, focusing on older adults’ daily routine, overall sensory appreciation, walking experience and overall satisfaction with the neighbourhood, which are then further discussed in reference to relevant local research in Section 4.

## 2. Materials and Methods

### 2.1. Study Areas

**Figure 1:**
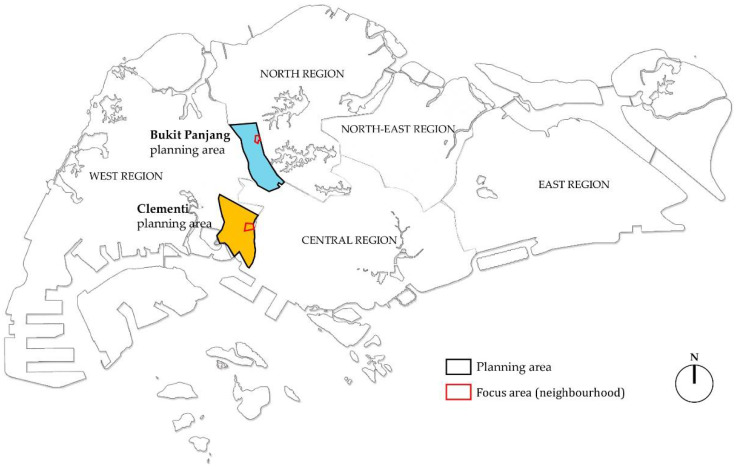
Location of study areas (source: by author).

Two typical high-rise, high-density public housing neighbourhoods in Singapore were selected for this study, situated in the Bukit Panjang and Clementi planning areas in Singapore’s West Region (Figure 1). As of 2019, 78.35% of Singaporeans lived in similar public housing neighbourhoods [11], which are built and operated by the Housing Development Board (HDB) since the 1960s. These so-called ‘HDB neighbourhoods’ are planned according to principles of modernist architecture, efficiency, cost-effectiveness, egalitarianism and multiculturalism and are highly regulated [94]. They represent high-quality living environments well equipped with a range of communal facilities and services. As such, Singapore’s public housing escaped the stigma often attached to social housing in some other parts of the world, such as the USA or the UK [95]. However, while targeted at low- and middle-income Singaporeans, public housing is not necessarily easily accessible for everyone. Single individuals, for instance, are eligible for public housing only at the age of 35 or above, and under specific monthly income levels [96]. Moreover, although they have transformed over time, HDB neighbourhoods are still sometimes described as homogenous and inauthentic, which Lai [97] found inadequate as she illustrated the complexity and multi-faceted identities of a local community in Marine Parade, one such neighbourhood. Neighbourhood spaces and amenities often represent the extension of people’s homes and are settings where various social activities take place. On the other hand, Pow [98] criticised the predominantly negative and pessimistic homogenous perception of private housing developments in Singapore, resulting from the public–private housing divide, and proposed a more ‘hopeful’ agenda to counteract it.

#### 2.1.1. Neighbourhood 1: Bangkit

**Figure 2:**
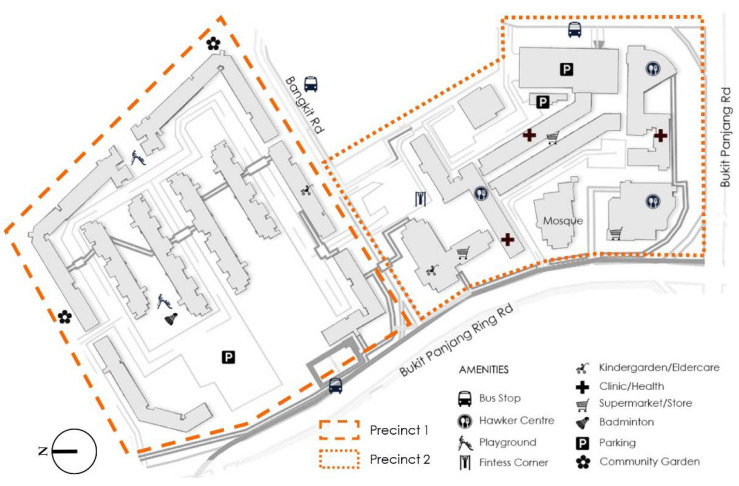
Neighbourhood 1: Bankgit, Bukit Panjang—spatial layout and amenities (source: by author).

**Figure 3:**
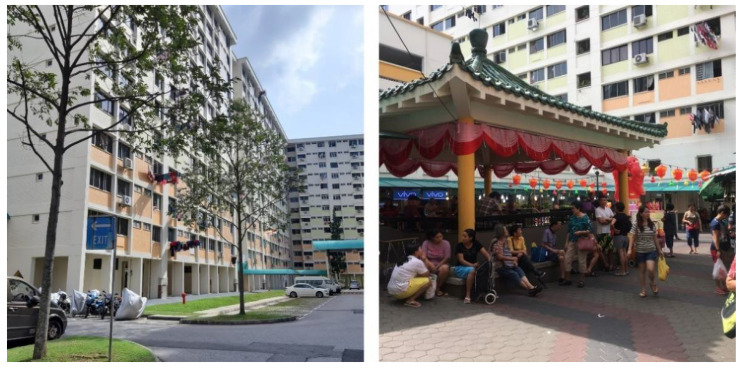
Bangkit neighbourhood: precinct 1 (left); precinct 2 (right) (source: by author).

The Bangkit neighbourhood is located within the Bangkit planning subzone of the Bukit Panjang planning area (Figure 2). In June 2020, the Bukit Panjang planning area had a total population of 138,270, with 35.61% of residents aged 50 and above, and 12.74% aged 65 and older [11]. A total of 83.77% of Bukit Panjang residents live in HDB apartments. The focus area, the Bangkit neighbourhood, comprises two precincts, with typical 12-storey slab blocks built in the 1980s, and an average gross plot ratio (GPR) of 2.8. One precinct (precinct 1) is predominantly residential, while the other (precinct 2) comprises a mix of residential and commercial land uses (including a wet market, eateries and a supermarket), as well as two places of worship and the town council (Figure 3). Other public amenities including children’s playgrounds, fitness corners, sport courts and community gardens are dispersed across both precincts. Underneath the housing blocks are the so-called ‘void decks’, which are multi-purpose public spaces typical for HDB buildings built since the 1980s.

#### 2.1.2. Neighbourhood 2: Clementi

**Figure 4:**
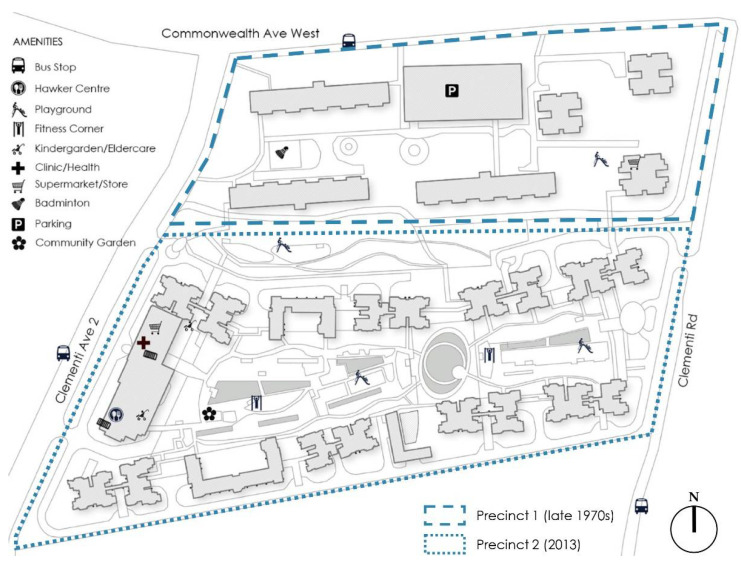
Neighbourhood 2: Clementi—spatial layout and amenities (source: by author).

**Figure 5:**
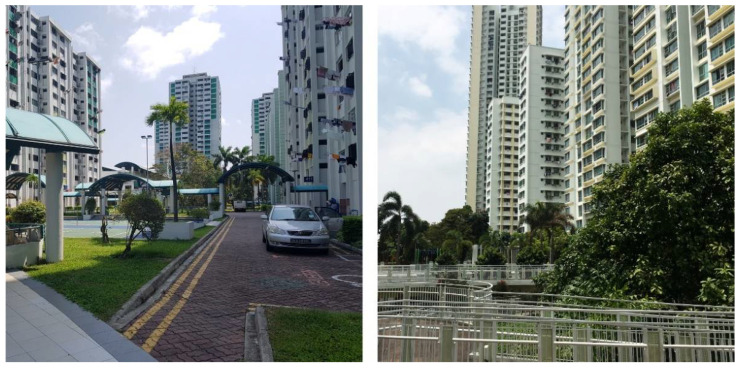
Clementi neighbourhood: precinct 1 (left); precinct 2 (right) (source: by author).

The Clementi neighbourhood is located in the Clementi Woods planning subzone of the Clementi planning area in Singapore’s West Region (Figure 4). As of June 2020, the Clementi planning area had a total population of 91,990, with 40.90% of residents aged 50 and above, and 20.24% aged 65 and older [11]. A total of 73.35% of Clementi residents live in HDB apartments. The main area of study includes two predominantly residential precincts (Figure 5), yet different in terms of building typology. The older precinct (precinct 1) was built in the late 1970s, with a gross plot ratio (GPR) of 2.8. It comprises three residential 24-storey tower blocks and three 12-storey slab blocks, similar to the Bangkit neighbourhood. Besides void decks, this older precinct also provides a children’s playground, badminton courts and pavilions. The newer precinct called Casa Clementi (precinct 2) was built in 2013, and it is considerably denser, with a GPR of 3.5. It comprises ten 40-storey blocks (without void deck spaces) surrounding an elevated landscaped deck on top of a 2-storey car park facility. The majority of public amenities (e.g., playgrounds, fitness corners, community garden, eldercare centre and kindergarten) are concentrated on this elevated deck, while some basic commercial amenities and a foodcourt are located at the ground level, along Clementi Avenue 2.

### 2.2. Exploratory On-Site Studies

Initial sensory explorations of the two sites were conducted together with 44 (24 + 20) student researchers who took the elective module ‘City and Senses: Multi-sensory Approach to Urbanism’ offered at the Department of Architecture, School of Design and Environment, National University of Singapore in AY2017/18 and AY2018/19. About half of the student researchers were international students who were less familiar with HDB neighbourhoods, which formed an important balance. Bodily engagement with the environment provided a common ground for all student researchers regardless of their gender, ethnic, cultural or educational background differences. On-site investigations included exploratory sensory photo-journeys and sensory notation exercises, followed by analysis of residents’ daily activity patterns and synthesis.

#### 2.2.1. ‘Sensing the Site’ Journeys

At the beginning of the study, teams of 3–5 student researchers were engaged in two twenty-minute photo-journeys to quickly familiarise themselves with the environment, capture their first subjective experience of the site and map their responses to sensory properties of the neighbourhood. These exercises were inspired by the Situationist technique of ‘dérive’ [99], which requires unplanned quick passage through an environment while dropping usual motivations for movement and allowing to be led by what is found and encountered. In the first journey, student researchers were asked to take the same team path and to each take 10 photographs of anything that attracted their attention along the way, using their mobile phone cameras. Each team traced the collective path on the map and marked the points where each photograph was taken. At the end of the walk, they described each photo taken using 3 keywords of their choice. In the second journey, teams took the same collective path again and made another set of 10 photographs, while focusing on documenting the key sensory properties of the neighbourhood and their subjective responses to such properties. Each photo was again described by 3 keywords and mapped. The two photo-journeys initiated embodied engagement with the neighbourhood and prompted comparisons and discussions about the subjectivity of multi-sensory experience and assessment, influenced by the socio-cultural background of researchers, the socio-cultural context of the investigated neighbourhood, familiarity with the site and/or local housing context, and climate and temporal factors. These factors informed the subsequent stages of research, including the selection of specific locations for notation of sensory and activity rhythms, and the specific sections of the socio-perceptual survey (phase 2).

#### 2.2.2. Documentation of Sensory Rhythms and Pedestrian Activity Patterns

Structured on-site observations were conducted by teams of student researchers at carefully selected points in the neighbourhood. As much as possible, fieldwork was conducted in an unobtrusive manner at points that allowed for a clear view of selected spaces and amenities, without direct contact with the residents. Observation points were informed by the insights from ‘sensing the site’ journeys, whereby the spaces photographed the most by the students during the two journeys were included. Additional points of observations included spaces near key neighbourhood amenities, such as bus or train stations, supermarkets, food centres, community centres, clinics, public plazas, playgrounds, community gardens or sports grounds. These additional points may have not been covered in the initial journeys, but they were considered important points of interest and generators of pedestrian activity in the neighbourhood.

Two types of observations, informed by the ‘snapshot’ technique proposed by Gehl and Svarre [100], were conducted. They included capturing sensory qualities and documenting pedestrian activity at each observation point in the neighbourhood, with an aim of identifying any patterns or relationships over time. They were conducted over short periods of time (5 min) and repeated three times—in the morning (8–10 a.m.), in the afternoon (1–3 p.m.) and in the evening (7–9 p.m.), during one workday and one weekend day, as well as under similar weather conditions (no rain).

Time of the week, time of the day and weather condition represent three critical factors for utilising neighbourhood spaces in Singapore. In general, there is a significant difference in the use of public spaces in Singapore during a workday and a weekend day, whereby weekends experience higher volumes and intensities. This is expected since the majority of working adults and school children are absent from the neighbourhood much of the time during workdays. However, this may not be the case for some neighbourhood spaces. Regardless of ‘business hours’, neighbourhood spaces, some of which are unsheltered, also tend to be utilised more intensively in the early mornings and in the late afternoons/evenings due to the local tropical climate, which is hot and humid, with high exposure to sun and frequent rain showers. Whether on workdays or on weekends, local residents tend to avoid spending time in outdoor spaces under the harsh sun, and the perception and the use of space considerably change after the sun goes down, which is typically around 7:20 p.m. Finally, observations in both Clementi and Bangkit neighbourhoods were conducted in late January and early February of 2018 and 2019, right before the Lunar New Year celebrations (16 February 2018, and 5 February 2019, respectively). Installed decorations at certain places in both neighbourhoods may have influenced the daily activity patterns of the residents and researchers’ assessment of multi-sensory properties, but not substantially.

*Sensory notation.* During on-site observations, student researchers explored various available tools to record and assess sensory experience systematically. The primary tool used was the sensory notation chart developed by Lucas and Romice [85] to capture and evaluate the presence, type and intensity of visual, aural, tactile, kinetic, thermal and chemical (smell) sensory properties of a place, each on a 5-point scale. This intuitive yet systematic tool provided means for uncovering the most dominant and the weakest sensory qualities at different points in the neighbourhood. The sensory notation chart does not address users’ responses to environmental stimuli, whether positive or negative, pleasant or unpleasant. In response, the tool was enriched by an additional scale capturing the level of comfort or pleasantness resulting from the recorded intensity of sensory stimuli. Each student researcher assessed both the intensity of sensory stimuli and the level of pleasantness individually using the same chart. In such a way, the sensory notion chart acted as an important means for articulating, comparing and negotiating subjective sensory experiences.

*Activity notation.* The focus of pedestrian activity documentation was on observing, noting down and counting the number of people passing by (engaged in transient activity) and performing stationary activities, whether passive (sitting, people watching, etc.) or active (exercising, playing, having a conversation, etc.), at each point of observation in the neighbourhood. Additional information about users’ gender, estimated age and ethnicity were also recorded, with the specific focus on senior residents’ movement and gathering points. Working in groups of 3–5 student researchers enabled observing different aspects simultaneously.

According to William H. Whyte [101], observations at peak hours can reveal considerably predictable patterns of use, while off-peak hours may provide better insights about users’ preferences, due to smaller crowds and greater choice in space utilisation. While the observations may have missed out some activities, as activity patterns may vary from day to day, this investigation offered empirical snapshot insights into the daily rhythms of local Singaporeans in two neighbourhoods. A similar method has been used in previous local studies, with similar activity patterns reported [102,103].

#### 2.2.3. Synthesis: Rhythm Analysis

The key ideas expressed in Henri Lefebre’s *Rhythmanalysis* [104] were employed to synthesise gathered sensory and activity data, and to uncover sensory and activity rhythms in the neighbourhood. This included identification of harmonious and conflicting conditions arising from specific spatial settings, sensory properties, sensory experiences and pedestrian activities. Selected neighbourhood places with such critical conditions were further investigated through design response explorations, some of which addressed the issues of wayfinding, safety, universal design, comfort, cultural practices, social inclusion, inter-generational interaction and immersive technologies, among others.

### 2.3. Socio-Sensory Perception Surveys

Socio-sensory perception surveys were conducted to gain insights into residents’ perception, satisfaction and utilisation of available spaces and amenities in their neighbourhoods from the perspective of sensory experience. The survey instrument was conceptualised based on insights gained from the exploratory on-site investigations. It comprises the following sections: (a) general information (age, gender, ethnicity, time lived in the neighbourhood, presence of any sensory impairment and self-reported overall health condition); (b) daily routine (frequency of going out, most frequently performed activities, most frequented, liked and disliked places in the neighbourhood); (c) overall sensory appreciation; (d) walking experience; and (e) overall satisfaction with the neighbourhood. See Appendix A for the full survey instrument (Table A1).

Specific factors discussed by the student researchers that influence one’s subjective sensory assessment (e.g., socio-cultural background, familiarity with the site and/or local housing context, climate and temporal factors) informed the general info section (a) of the survey. Observing activity and sensory patterns informed the survey questions in section (b) daily routine activities (Q2–Q8). Initially identified themes/issues through rhythm analysis informed several questions, e.g., Q10c (culture), Q11a and Q11b (safety and universal design), Q11d (wayfinding), Q11g (climate) and 12b (inter-generational interaction).

All surveys were conducted on site. The majority of survey participants were recruited with the support of the Holland-Bukit Panjang Town Council in Bukit Panjang and the Lions Befrienders Senior Activity Centre in Clementi, who invited the research team to join several activities and gatherings of older adults that they organised or knew about. These partners were identified through on-site observations and analysis. A small number of survey participants were recruited at places in the neighbourhood where higher concentrations of elderly residents have been observed. Surveys were distributed in either English or Mandarin, depending on participants’ preference. A researcher speaking English, Mandarin and a local dialect assisted the participants who needed further explanation of any question or to fill in the questionnaire on their behalf.

#### Statistical Analysis

Statistical analysis was conducted in IBM SPSS Statistics 25. A non-parametric ‘Spearman rank-order correlation coefficient’ was employed to measure the strength and direction of association that may exist between specific variables measured on at least an ordinal scale. The strength of association is shown by the correlation coefficient (*r*) and measured by the coefficient of determination (*r*2). While there are different interpretations of the correlation coefficient (*r*) (see, e.g., [105,106,107]), this study adopts Parker’s classification [108], whereby the strength of relationship can be interpreted as ‘very strong’, when the (*r*) value is between 0.80 and 1.00, ‘strong’ (*r* = 0.60–0.79), ‘moderate’ (*r* = 0.40–0.59), ‘weak’ (*r* = 0.20–0.39) and ‘very weak’ (*r* = 0.00–0.19). The level of significance of the association is indicated by the probability level (*p*-value), e.g., statistically significant at *p* = 0.05, which expresses the likelihood of a certain correlation coefficient (*r*) to occur by chance. A smaller *p*-value indicates a more significant relationship.

A non-parametric ‘Mann–Whitney U test’ was also used to check if there were any statistically significant differences in socio-sensory assessment between participants who reported any sensory impairment and those who did not.

Statistical analysis was performed for the two neighbourhoods separately, given the considerable difference in the number of survey participants in Bangkit and Clementi. Moreover, although representing typical HDB neighbourhoods planned according to established common guidelines, the two neighbourhoods differ in several aspects, such as date of construction, spatial arrangement, building typology, density, and number and arrangement of neighbourhood amenities, among others, which makes the analysis of a combined sample challenging. Finally, due to the small sample size in Clementi (*n* = 66), the following Section 3 (particularly Section 3.2.3, Section 3.2.4, Section 3.2.5 and Section 3.2.6) focuses primarily on correlations investigated in the Bangkit neighbourhood, with only indicative findings provided for Clementi.

## 3. Results

### 3.1. Rhythm Analysis Findings

**Figure 6:**
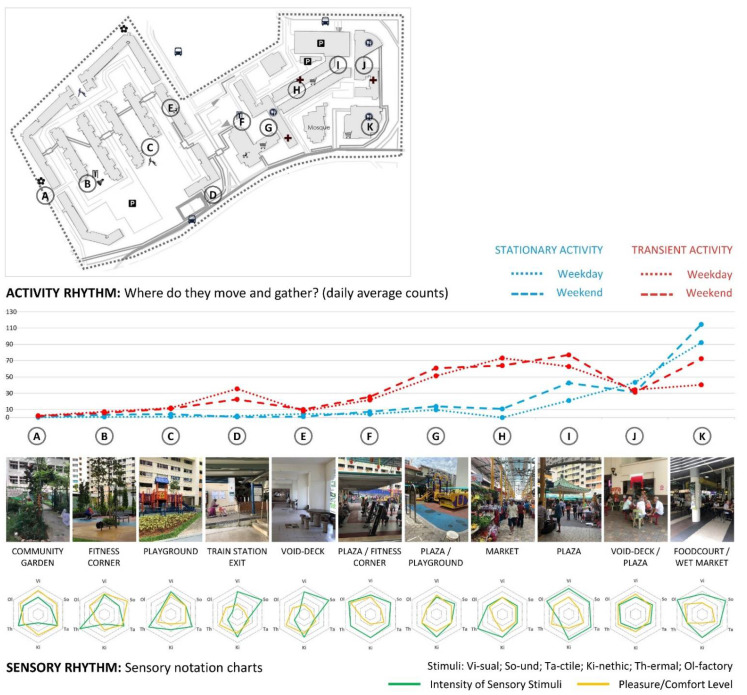
Summary of rhythm analysis in Bangkit neighbourhood (source: by author).

**Figure 7:**
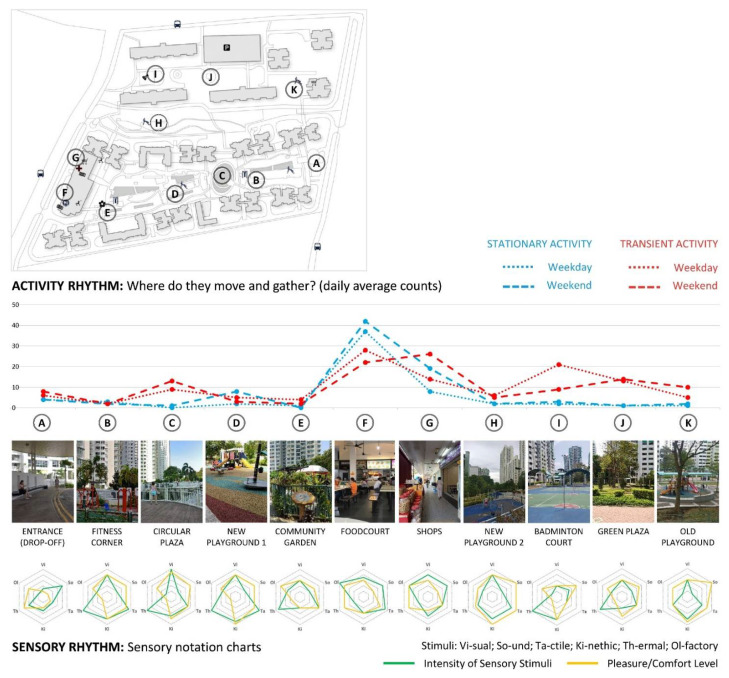
Summary of rhythm analysis in Clementi neighbourhood (source: by author).

Findings from the initial on-site analysis and synthesis performed by the student researchers show that both neighbourhoods are the most active in the morning and the late afternoon/evening, when the local tropical climate conditions (sun and heat) are the most conducive for pedestrian activity. Both neighbourhoods show to be overall more intensively used during the weekend, especially for stationary activities. Transient activities predominate, particularly around commercial areas and sheltered walkways, which connect key facilities in the neighbourhood with the nearest public transportation points. Foodcourts and commercial areas are the places of the most intense stationary activities amongst senior residents (see Figure 6 and Figure 7 for the summaries of rhythm analyses in Bangkit and Clementi, respectively).

Sensory notation analysis shows that places around eateries and shopping activities are also often the richest in overall sensory quality (visual, sound, olfactory and tactile). However, rich sensory stimulation does not necessarily reflect the overall quality of a place, as the quantity, intensity and complexity of multi-sensory stimuli can be both a barrier and a facilitator to older adults’ active spatial and social engagement. For example, a fitness corner and a playground in Clementi (see ‘B’ and ‘D’ in Figure 7) have both been evaluated as rich in terms of visual, tactile, kinetic and thermal experience, and overall pleasant. However, the lack of shelter contributes to high exposure to sun and low thermal comfort, which results in this place not being used during the day. Moreover, the lack of seats that would overlook the playground does not encourage interaction between different generations. A missed opportunity for inter-generational bonding is also represented by the lack of conscious integration between the eldercare facility, fitness corner, playground and community garden, despite good intentions to locate them next to each other.

On the other hand, the void deck space next to the plaza, wet market and foodcourt in the Bangkit neighbourhood (see ‘J’ in Figure 6) was evaluated by the students as only moderately rich in all sensory stimuli, and overall moderately pleasant. However, this is one of the main places where older adults like to linger, despite it being somewhat messy and without seats provided. In fact, many older residents bring their own plastic chairs to sit in this sheltered place, which allows them to be at the centre of the neighbourhood’s daily life throughout the day and under any weather condition.

Besides comfort and inter-generational bonding, several other topics arose from the rhythm analysis, which are related to sensory experience and ageing-friendly design, including safety, universal design, wayfinding, cultural practices and social inclusion. Several conditions have been identified as potentially dangerous for walking, due to numerous obstacles, such as steps, curbs, uneven surfaces and slippery and contrasting floor surfaces. While barrier-free pathways are generally provided, it was observed that these are rather inconvenient and sometimes complicated or too long (compared to other paths) and limited the choice of walking routes. Moreover, it was also observed that many of the seating amenities did not have backrests or armrests to support older users in sitting down and standing up. Some of the handrails along ramps or stairs did not have a round shape and thus were difficult to grip. Parts of both neighbourhoods were perceived as somewhat visually homogenous, with signage not always well positioned, and thus difficult to orientate within. Students also observed traces of cultural practices at some areas in the neighbourhood, such as incense burning or offerings, as well as areas that looked messy and neglected, which may have impacts on residents’ perception and use of these spaces. These initial findings reflect the perspective of young adults (student researchers) who do not live in any of the two neighbourhoods or visit them frequently. They all contributed to the formulation of several survey questions, as indicated in Section 2.3.

### 3.2. Survey Results

#### 3.2.1. Survey Sample and General Information

The sample size included a total of *n* = 301 adult survey participants, 235 of whom were from Bangkit and 66 from the Clementi neighbourhood.

*Gender profile.* The majority of survey participants in Bangkit were female (almost 60%), while in the Clementi neighbourhood, there were more male participants (over 54%) (Table 1).

*Age profile.* The majority of survey participants in both neighbourhoods (Bangkit—71%; Clementi—81.8%) were at least 50 years of age (Table 1). Based on physical and mental ability and health condition, senior adults are often categorised as: (a) the ‘Young-Old’ (ages 65–74)—physically and mentally fit and fully functioning; (b) the ‘Old’ (ages 75–84)—considerably independent or semi-independent, requiring some level of assistance; and (c) the ‘Oldest-Old’ (aged 85 and older)—requiring a high level of medical care (see, e.g., [109,110]). Bozovic-Stamenovic [111] expanded this classification to include the so-called (d) ‘Oldish’ adults, referring to a borderline category of adults aged 50–64, who are mobile and active, yet ‘becoming old’ and with new aspirations that are often neglected in neighbourhood design. Participants in this study were initially classified according to these four age groups. However, since only four survey participants (one in Bangkit and three in the Clementi sample) belonged to the ‘Oldest-Old’ category, these participants were subsequently merged into the ‘Old’ group (75 and over). All other participants were categorised as ‘Adult’ (ages 18–49). Participants younger than 18 were not included in accordance with NUS-IRB approval.

*Ethnicity profile.* As of June 2020, Chinese represented 74.3% of the total resident population in Singapore, followed by Malays (13.5%), Indians (9%) and others (3.2%) [10]. The survey sample in this study does not reflect such ratios, with Chinese comprising almost 90% of the survey participants in both neighbourhoods (Table 1). As a result, ethnicity was not considered in the following statistical analysis.

*Time lived in the neighbourhood.* The majority of residents in both neighbourhoods lived there for at least 5 years (see Supplementary Materials, Table S1). A total of 77% of residents lived in Bangkit, Bukit Panjang, for 20 years or longer. On the other hand, 71.9% of survey participants stayed in Clementi between 5 and 19 years, which reflects the fact that the new precinct (Casa Clementi) was built in 2013, with the majority of survey participants coming from this precinct. Time lived in the neighbourhood was not considered in the following statistical analysis.

*Reported sensory impairments.* Over 25% of survey participants in both neighbourhoods reported at least one sensory impairment (Table 2). As expected, the number of reported sensory impairments increases with age (Table 3). The most prominent sensory decline reported in both neighbourhoods was poor vision, followed by walking difficulties/motor decline and poor hearing (Table 4).

*Reported health condition.* Survey participants reported their health condition using a five-point scale, whereby (1) stands for a ‘poor’ health condition and (5) refers to an ‘excellent’ health condition. Over 16% of participants in Bangkit and over 32% in Clementi reported ‘poor’ (1) or ‘could be better’ (2) health conditions (Table 5). The average self-reported health condition is good, which is reflected in the mean scores of 3.31 and 3.05 (out of 5.00) for Bangkit and Clementi, respectively. The scores vary across different age groups (Table 6).

As anticipated, health condition scores decrease with age increments. The Spearman correlation test confirmed a statistically significant yet weak negative correlation between the reported health condition score and age increments in the Bangkit neighbourhood sample (*r* = −0.297; *p* = 0.000) (see Supplementary Materials, Table S2). Moreover, the Mann–Whitney U test results indicate that having any sensory impairment significantly impacts the self-assessment of one’s overall health condition. Specifically, Bangkit residents with any sensory impairment tend to report a poorer health condition (U = 4668.500; *p* = 0.000) (see Supplementary Materials, Table S3).

#### 3.2.2. Daily Routine

*Frequency of going out.* Over 15% of participants in both neighbourhoods indicated a low frequency of going out, i.e., less than once a day (Table 7).

While the frequency of going out varies across age groups, no statistically significant correlation was found between age increments and decreased frequency of going out. However, the Spearman correlation test revealed a statistically significant yet weak negative correlation (*r* = −0.239; *p* = 0.000) between better self-reported health condition and higher frequency of going out, in the Bangkit neighbourhood sample (see Supplementary Materials, Table S4). In other words, residents who reported better health tend to go out more frequently. The Mann–Whitney U test did not reveal any significant difference in the frequency of going out between participants with and without any sensory impairment.

*Predominant activities in the neighbourhood.*Table 8 summarises the most frequently performed activates in the two neighbourhoods, as reported by the residents (regardless of age), which include shopping, eating, meeting friends, commuting and strolling. No significant difference was found among different age groups. However, in both neighbourhoods, commuting is overall more prevalent among younger adults, while older adults tend to stroll around, meet friends, visit community organisations and engage in gardening and exercise activities more frequently than younger residents (see Supplementary Materials, Tables S5 and S6). Such findings largely align with the initial insights from the rhythm analysis.

*Important spaces in the neighbourhood.* The most *frequently visited places* in both neighbourhoods (regardless of age) are shopping spaces, eateries and community organisations (see Supplementary Materials, Table S7), which is in line with the rhythm analysis findings. In addition to these spaces, residents of the Bangkit neighbourhood also frequently visit the market (34.3%) and parks and gardens (21.2%). On the other hand, the Clementi neighbourhood does not offer any large park amenities and markets. Except for the shopping spaces, which are dominated by the younger adults, all other neighbourhood amenities are more frequently visited by the older adults (aged 50 and above) (see Supplementary Materials, Tables S8 and S9). Frequented places are typically described by the residents as convenient, relaxing, comfortable and conducive for social interaction and as spaces that boost positive emotions (see Supplementary Materials, Figures S1 and S2).

The residents reported similar patterns in respect to most *liked places* in the neighbourhood (see Supplementary Materials, Table S10). Younger adults (age 18–49) tend to like shopping spaces somewhat more than other age groups, while older adults (age 50 and above) tend to prefer markets and community organisations more than younger adults. Eateries, parks and gardens are somewhat equally appreciated by all age groups in both neighbourhoods (see Supplementary Materials, Tables S11 and S12, Figures S3 and S4).

The majority of residents in both neighbourhoods reported the local coffee shops and parks and gardens as the main *meeting places* where they usually socialise with their friends and neighbours. Alternative meeting venues include shopping spaces and community organisations, as well as other places such as residents’ homes (see Supplementary Materials, Tables S13–S15).

Over 70% of all participants (regardless of age) reported messy or dark areas close to rubbish collection points as the most *disliked places* in the neighbourhood. Interestingly, these areas are followed by the hawker centres despite also being among the most frequented and liked neighbourhood amenities, as well as important meeting venues (see Supplementary Materials, Tables S16–S18). The most disliked places were most directly associated with negative sensory experience, including unpleasant smell, noise, crowdedness, dirtiness and inconvenience (see Supplementary Materials, Figures S5 and S6).

#### 3.2.3. Overall Sensory Appreciation

Residents found both neighbourhoods overall very good in terms of aesthetic appeal (Bangkit: 3.87; Clementi: 3.88), cleanliness (Bangkit: 3.86; Clementi: 3.42), variety of distinguishable ambiences (Bangkit: 3.76; Clementi: 3.52) and availability of features representing different cultures (Bangkit: 3.63; Clementi: 3.52). However, significantly lower appreciation was found towards smell, noise, overall sensory bombardment in the neighbourhood and crowdedness (Table 9).

A considerable proportion of residents in both neighbourhoods ‘strongly agreed’ (5) or ‘somewhat agreed’ (4) that spaces around their homes were occasionally too crowded, noisy, smelly and overwhelming in terms of senses on an everyday basis. Dissatisfaction with crowdedness (37.96%) and noise (24.43%) levels was slightly higher in the Bangkit neighbourhood (see Supplementary Materials, Table S19), while the cleanliness level (26.16%), smell (24.62%) and sensory bombardment (21.54%) were slightly more disliked among Clementi residents (see Supplementary Materials, Table S20).

*Relationship between sensory appreciation, age, reported health condition and sensory impairment.* Spearman correlation analysis results indicate that certain aspects of overall sensory appreciation may be associated with age and health condition. However, only very weak statistically significant correlations were found (Table 10). For instance, the analysis across the Bangkit sample shows that older adults tend to be less negatively affected by the crowd level than younger adults (*r* = −0.138; *p* = 0.048). Moreover, across the same sample, healthier adults tend to rate the neighbourhood’s cleanliness level (*r* = −0.139; *p* = 0.044), smell (*r* = −0.180; *p* = 0.009), noise (*r* = −0.178; *p* = 0.010) and sensory bombardment (*r* = −0.198; *p* = 0.004) more positively than those who reported poorer health.

The Mann–Whitney U test did not show any significant difference in overall sensory appreciation between participants with and without any sensory impairment.

#### 3.2.4. Walking Experience

Residents found their neighbourhoods overall good in terms of walking experience. The residents particularly appreciated walkways surrounded by greenery (Bangkit: 4.06; Clementi: 3.94) and sheltered pathways (Bangkit: 4.15; Clementi: 3.73) as the key conditions that make the walking experience more enjoyable. This was expected given the local tropical climate. However, they showed lower appreciation for conditions related to walking safety (Table 11).

A substantial percentage of residents ‘strongly agreed’ (5) or ‘somewhat agreed’ (4) that their neighbourhoods are unsafe due to too many obstacles (Bangkit: 30.55%; Clementi: 25.76%) and slippery floors (Bangkit: 34.29%; Clementi: 51.51%), especially when it rains. Moreover, a significant percentage of residents reported that they hesitate to go out without someone accompanying them (Bangkit: 12.81%; Clementi: 15.48%) or sometimes have troubles finding their way in the neighbourhood (Bangkit: 10.68%; Clementi: 15.16%) (see Supplementary Materials, Tables S21 and S22). These are the critical barriers to a safe and enjoyable walking experience. These findings align with the initial rhythm analysis insights.

*Relationship between walking experience, age, reported health condition and sensory impairment.* Correlation analysis findings indicate some potential relationships between certain aspects of walking experience and age and health condition. Very weak and weak statistically significant correlations were found in the Bangkit neighbourhood sample (Table 12). Older adults living in Bangkit tend to hesitate to go out (*r* = 0.255, *p* = 0.000) or get lost (*r* = 0.147, *p* = 0.040) in their neighbourhood more than younger adults. On the other hand, younger and healthier adults in Bangkit tend to appreciate nature more than older residents (*r* = −0.191, *p* = 0.007) and those who reported poorer health (*r* = 0.158, *p* = 0.026). Finally, adults with poorer health tend to agree with all the statements pertinent to a negative walking experience (too many obstacles, slippery floor, hesitation to go out and wayfinding problems) more than those reporting good health.

The Mann–Whitney U test did not show any statistically significant difference in the assessment of walking experience between participants with and without any sensory impairment.

#### 3.2.5. Overall Satisfaction with the Neighbourhood

Overall, residents seem to be very satisfied with living in their respective neighbourhoods, with very similar scores across both neighbourhoods (Table 13).

Only 8.50% of Bangkit residents and 10.94% of Clementi residents expressed that their neighbourhood did not offer enough opportunities for inter-generational socialisation. However, while only 6.00% of Bangkit residents found that their neighbourhood was not elderly-friendly, 15.16% of Clementi residents felt the same (see Supplementary Materials, Tables S23 and S24).

*Relationship between overall satisfaction with the neighbourhood, age, reported health condition and sensory impairment.* Correlation analysis results show that overall satisfaction with the neighbourhood may be related to health condition, but not to age (Table 14). Very weak and weak correlations suggest that healthier adults tend to be satisfied with the neighbourhood amenities (Bangkit: *r* = 0.151, *p* = 0.034) and the neighbourhood’s elderly-friendliness (Bangkit: *r* = 0.262, *p* = 0.000) more than those who reported poorer health. Healthier residents also tend to feel happier living in the neighbourhood than those with poorer health (Bangkit: *r* = 0.213, *p* = 0.003).

The Mann–Whitney U test did not show any statistically significant difference in overall satisfaction with the neighbourhood between participants with and without any sensory impairment.

#### 3.2.6. Other Correlations

Additional analysis was employed to explore any significant correlations between different aspects of sensory assessment, walking experience and overall satisfaction with the neighbourhood. This analysis was conducted for the Bangkit neighbourhood only. The analysis found a considerable number of weak and moderate statistically significant such correlations. Table 15 highlights only moderate correlations (see Supplementary Materials, Table S25, for all other correlations).

Several observations can be made based on such moderate correlations.

*Aspects of sensory appreciation are interconnected.* Adults who find their neighbourhood noisy also tend to find it smelly (*r* = 0.447, *p* = 0.000). Negative smell assessment is also related to negative assessment of sensory bombardment (*r* = 0.437, *p* = 0.000). The availability of distinguishable ambiences tends to contribute to a higher aesthetic appeal of the neighbourhood (*r* = 0.422, *p* = 0.000).

*Spatio-sensory qualities of a neighbourhood influence the perception of safety and walking autonomy.* Residents who find their neighbourhood having too many obstacles for walking tend to find it also having slippery floors (*r* = 0.595, *p* = 0.000). Moreover, adults who sometimes get lost in their neighbourhood also tend to hesitate to go out without someone accompanying them (*r* = 0.505, *p* = 0.000).

*Satisfaction with neighbourhood amenities is related to assessment of elderly-friendly design, inter-generational social interaction and satisfaction with living in the neighbourhood (happiness)*. Adults who are more satisfied with the amenities in their neighbourhood tend to also agree that there are plenty of opportunities for inter-generational interaction (*r* = 0.483, *p* = 0.000), and that their neighbourhood is well designed for the elderly residents (Bangkit: *r* = 0.438, *p* = 0.000). Residents satisfied with neighbourhood amenities also tend to feel happier living in the neighbourhood (*r* = 0.498, *p* = 0.000). Finally, residents who tend to agree that their neighbourhood design is elderly-friendly also tend to feel happier living in that neighbourhood (Bangkit: *r* = 0.427, *p* = 0.000).

## 4. Discussion

In a recent review of research focusing on age-friendly neighbourhoods in Singapore, Yuen and colleagues [112] observed that such research is in its nascent stage, with very limited studies investigating older adults’ everyday lived experience and perception of their built environment. According to Bhuyan and colleagues [113], most of such studies focused on either the physical or the social environment, with only limited research investigating both dimensions concurrently and holistically. Only a handful of these studies included some yet limited aspects of older adults’ perception and sensory assessment as part of larger investigations. Cao and colleagues [114], for instance, included an aesthetics category (nature, buildings, noise and cleanliness) to understand the out-of-home behaviour of older Singaporeans. In their study, Gan, Fung and Cho [115] proposed a holistic assessment of ‘Older People’s Neighborhood Experience’, which captured some elements of environmental pleasantness and outdoor aesthetics, such as appreciation of greenery, unique neighbourood features and different ambience qualities. Similarly, Hou and colleagues [116] considered both objective and subjective (perceived) measures of built environments in reference to older adults’ travel/walking behaviour, whereby perceived measures included those on aesthetics, cleanliness and safety.

Through socio-sensory assessment of two local housing neighbourhoods, this exploratory study contributes to a better understanding of the relationship between multi-sensory qualities of neighbourhood spaces and multi-sensory experience, walking experience, overall satisfaction with the neighbourhood, age and self-reported health condition. In such a way, this study also provides insights about the role and capacity of a multi-sensory approach to informing the design of ageing-friendly neighbourhoods.

The survey findings, in conjunction with rhythm analysis, indicate that everyday movement and outdoor activity patterns of older adults, including frequency of going out and choice of neighbourhood spaces, may depend on numerous factors, such as the presence, quality and spatial arrangement of public amenities and walking infrastructure, and subjective assessment of sensory qualities of neighbourhood spaces, including comfort, safety, hygiene and aesthetic appeal, among others. The findings suggest that some of these factors are associated with both age and self-reported health condition. Such results correspond to some recent local studies which observed the connection between environmental conditions, going out and outdoor behaviour, and physical, psychological and social well-being of elderly residents (e.g., [115,116,117,118,119]). For instance, in their qualitative study, Bhuyan and Yuen [120] concluded that older adults tend to associate safety and pedestrian-friendly features with physical health, public amenities with social well-being, and aesthetic appeal and wayfinding with mental health.

Daily spatial practices in Singapore’s HDB neighbourhoods are heavily influenced by the new town spatial configuration (since the 1970s and 1980s), which assumes a hierarchy of spatial, activity and movement opportunities branching out from one’s home, apartment block and precinct towards the neighbourhood centre and town centre [121]. In such a system, a person encounters a linear progression of spaces and amenities, from the more intimate ones closer to home, such as void decks, recreational nodes (e.g., playgrounds, sports and exercise areas), community gardens, coffeeshops and convenience stores, to the more public amenities closer to the town centre, such as foodcourts, markets, schools, healthcare facilities and train stations, among others. It is nearer the home where more spontaneous and intense social interactions are most likely to occur, whereby different user groups tend to utilise places in different patterns and frequencies, depending on time of the day or the week, weather conditions, personal interests and abilities.

The survey findings indicate that 17.5% of all surveyed participants go out less than once per day. This is in line with the study by Wu and Chan [122], which showed that 21.9% of Singaporean older adults did not participate in neighbourhood activities on a daily basis, which may also be a predictor of social isolation. Moreover, survey analysis shows that the frequency of going out is associated with a better self-reported health condition, which aligns with other studies asserting that engaging with everyday outdoor activities is critical for older adults’ quality of life and independence. For example, research by Niti and colleagues [123] showed that performing everyday productive activities (such as buying groceries, preparing meals, community work or gardening) may lower the risk of cognitive decline significantly more than physical or social activities. It is important to note, however, that many productive activities may also include elements of both physical and social engagement. A study by Chong and colleagues [124] also concluded that older Singaporeans who were more satisfied with neighbourhood amenities and outdoor facilities used them more often and tended to feel less depressed while having a stronger sense of community cohesion.

The survey results and activity mapping show that the most frequently visited places in both Bangkit and Clementi are shopping spaces, eateries and community organisations, with strolling, shopping, eating out and meeting friends being the predominant activities among older Singaporeans. According to Yuen and colleagues [125], these are also the activities for which the frequency of participation declines with age. On the other hand, the frequency of participation in meeting friends at the void deck spaces and religious activities shows a trend of an increase with age. Studies have shown that accessibility to open spaces, parks, recreational facilities, eateries, markets, shops and train stations and their presence along safe and comfortable pedestrian pathways enable outdoor physical and social activities among older Singaporeans [117], increase the frequency of going out [116] and thus contribute to their physical and mental health. Research by Hou and colleagues [116], for instance, indicated a significant positive association between the proximity to wet markets and an increased tendency in residents to walk more frequently. On the other hand, a study by Tao and colleagues [118] suggested that a greater number of nearby amenities significantly decreases the average walking time and daily activity levels of older adults, which calls for more careful planning of neighbourhood facilities to promote physical activity among the elderly residents.

Besides public amenities, the results in this study reveal important insights into how specific sensory assessments influence older adults’ daily activities and behaviour in two neighbourhoods, both positively and negatively. The survey findings show overall high appreciation of neighbourhoods’ aesthetic appeal, particularly natural elements, hygiene and cleanliness, ambient diversity and presence of features pertinent to different cultures. Statistical analysis also indicates that more positive assessments of cleanliness, sound level, smell and sensory bombardment levels are associated with a higher self-reported health condition. Furthermore, the higher assessment of aesthetic appeal might be associated with higher satisfaction with neighbourhood and public amenities, better perception of age-friendly design and lower hesitation to go out. In many instances (i.e., elderly-friendliness, satisfaction with amenities and living in the neighbourhood, aesthetics, noise, cleanliness, nature, safety and overall walking experience), healthier adults tend to assess their everyday outdoor environment more positively. This suggests that the sensory experience of older adults should be given greater attention.

According to Cao and colleagues [114], the presence of nature, as well as quiet well-maintained spaces, encourages older adults to go out. However, correlation analysis in this study did not find any such statistically significant associations. On the other hand, a considerable number of surveyed residents found their neighbourhood unsafe due to too many obstacles (level changes), slippery floors and a lack of clear and intuitive wayfinding features. In fact, correlation analysis shows that lower aesthetic appeal, poor hygiene and maintenance, poor assessment of smell and sound, walking obstacles, slippery floors and poor wayfinding tend to relate to residents’ hesitation to go out independently. While the presence of sheltered walkways and barrier-free pathways may enable safe and comfortable movement through the neighbourhood, untidy and dark areas, typically found at the ‘back lanes’ near rubbish collection points and associated with an unpleasant smell, noise and crowd, are often avoided. This further limits older adults’ choice of walking paths in the neighbourhood and shrinks the already confined movement sphere due to lower mobility.

The survey findings align with observations on site that much of the neighbourhood design to support the elderly, including sheltered walkways, handrails, ramps, seats and exercise areas, tends to be only elementary and rather utilitarian, driven by a ‘do no harm’ principle and with a lack of attention to details and finishing. Such design reveals missed opportunities for greater integration between pragmatic functions and design qualities capable of boosting pleasure, curiosity, meaningful engagement with space and other users and even challenging one’s physical and mental abilities.

*Limitations.* Some challenges and limitations to this exploratory study should be acknowledged, which primarily arise from the lack of empirical methods to capture and assess subjective multi-sensory experience. The initial sensory notations were conducted by two different groups of young adults (graduate student researchers) who were not familiar with the neighbourhoods. The findings, thus, may not reflect the perspective of local and, in particular, older residents, as also proven in some aspects through the survey. Although systematic, the sensory notation charts employed at the initial stage of this study remain subjective, despite the aggregated assessment of several researchers. They were, however, helpful for relative comparisons, obtaining the initial understanding of sensory–spatial and activity rhythms in the neighbourhood and informing the survey instrument.

The sample sizes for the survey conducted in the two neighbourhoods are substantially different, with the sample of the Clementi neighbourhood being very small for some segments of the statistical analysis. Moreover, although the selected sites represent typical public housing neighbourhoods in Singapore, they do not fully represent the diversity of local urban environments and communities. The two neighbourhoods are different on several dimensions, ranging from the spatial typology and density to the number and spatial arrangement of public amenities, which makes the findings rather site-specific and difficult to generalise. While the study covered older adults of different age ranges, ethnicities, sensory impairments and health conditions, the proportions were not balanced or representative, and due to small sample sizes, the findings reported in this article cannot be generalised and are only indicative. Moreover, the results of the statistical analysis do not indicate any causalities, but only associations. Future research would be needed to examine any causal relationship between socio-sensory appreciation of built environments and health. Moreover, this study only measured self-reported health. Further study should involve more objective measures of health, using established instruments and scales, as well as other available mechanisms. According to Bozovic-Stamenovic [126], the obstacle in measuring the healthfulness of a neighbourhood is the lack of comprehensive and holistic evidence-based standards and guidelines. While it may be relatively easy to assess the accessibility of a neighbourhood space using available checklist instruments, typically based on universal design principles, these instruments often fail to address all facets of healthfulness, which encompass physical, psychological and social well-being, and require greater in-depth analysis from micro-, meso- and macro-perspectives and subjective experience simultaneously and in an integrated manner.

Although the study employed several methods for capturing and assessing multi-sensory qualities and subjective sensory experience, the research would benefit from gathering and triangulating more in-depth data, both quantitative and qualitative. In fact, the subsequent pilot study has been conducted in the Clementi neighbourhood with four senior residents [127]. This small study employed a combination of eye tracking to gather empirical data about older adults’ visual attention in the real-world environment and post-walk interviews to supplement visual information with qualitative narratives regarding other non-visual senses and embodied spatial experience. Rhythm analysis played a more explicit role in this study as a conceptual framework for integrating and interconnecting measured and experienced, quantitative and qualitative multi-sensory data and for gaining a more comprehensive and holistic understanding of older adults’ perception and multi-sensory experience of their neighbourhood.

## 5. Conclusions

This exploratory study has shown that the sensory richness of a neighbourood can affect the way older residents appreciate and utilise their everyday outdoor environments, and eventually their overall sense of well-being. However, sensory richness does not necessarily assume quality or a healthful experience, as it can present both an enabler and a barrier. We are yet to explore the full capacities of a balanced, strategic and smart manipulation of multi-sensory stimuli, largely neglected in empirical architectural and age-supportive design research, as potentially powerful enhancers of environmental quality and character, joyful and meaningful urban experience, intuitive triggers of positive human behaviour and active components of holistic care, social interaction and integration.

## Figures and Tables

**Table 1:** Survey sample: gender, age and ethnicity profiles.

	Bangkit		Clementi	
**Gender**	***n***	**%**	***n***	**%**
Male	94	40.2	36	54.5
Female	140	59.8	30	45.5
Total Valid	234	100.0	66	100.0
Missing	1		0	
Total	235		66	
**Age**	***n***	**%**	***n***	**%**
Adult (18–49)	65	29.0	12	18.2
Oldish (50–64)	73	32.6	13	19.7
Young-Old (65–74)	56	25.0	24	36.4
Old (75+)	30	13.4	17	25.8
Total Valid	224	100.0	66	100.0
Missing	11		0	
Total	235		66	
**Ethnicity**	***n***	**%**	***n***	**%**
Chinese	211	90.2	58	87.9
Malay	10	4.3	4	6.1
Indian	12	5.1	2	3.0
Other	1	0.4	2	3.0
Total Valid	234	100.0	66	100.0
Missing	1		0	
Total	235		66	

**Table 2:** Reported sensory impairments (regardless of age).

	Bangkit		Clementi	
	*n*	%	*n*	%
One sensory impairment	47	20.0	18	27.2
Two or more sensory impairments	12	5.1	4	6.1
Any sensory impairment	59	25.1	22	33.3
No sensory impairment	176	74.9	44	66.7
Total	235	100.0	66	100.0

**Table 3:** Reported sensory impairments across age groups.

	Bangkit				Clementi			
	*n* (S.Im)	% of Total S.Im	*n* (A.Gr)	% of A.Gr	*n* (S.Im)	% of Total S.Im	*n* (A.Gr)	% of A.Gr
Adult (18–49)	3	5.5	65	4.6	4	9.1	12	16.7
Oldish (50–64)	19	34.5	73	26.0	5	22.7	13	38.5
Young-Old (65–74)	18	32.7	56	32.1	9	40.9	24	37.5
Old (75+)	15	27.3	30	50.0	6	27.3	17	35.3
Total (S.Im.)	55	100.0	224	24.5	22	100.0	66	33.3
Missing			11				0	

S.Im—sensory impairment; A.Gr—age group.

**Table 4:** Reported specific sensory impairments.

	Bangkit			Clementi		
	*n*	%	% of Cases ^1^	*n*	%	% of Cases ^1^
Poor Vision	34	43.4	54.1	13	46.4	56.5
Poor Hearing	19	25.0	31.1	4	14.3	17.4
Poor Smell Detection	3	3.9	4.9	0	0.0	0.0
Walking Difficulties	19	25.0	31.1	10	35.7	43.5
Cognitive Difficulties	0	0%	0.0	1	3.6	4.3
Other	2	2.6	3.3	0	0.0	0.0
Total Answers	76	100.0	124.6	28	100.0	121.7
Total Cases	61			23		

^1^ As this was a multiple-answer question, total percentage does not equal 100%.

**Table 5:** Reported health condition.

	Bangkit		Clementi	
	*n*	%	N	%
Poor (1)	5	2.2	1	1.5
Could be better (2)	32	14.2	20	30.8
Good (3)	108	48.0	24	36.9
Very good (4)	45	20.0	15	23.1
Excellent (5)	35	15.6	5	7.7
Total Valid	225	100.0	65	100.0
Missing	10		1	
Total	235		66	

**Table 6:** Health condition scores across age groups.

	Bangkit			Clementi		
	Mean	*n*	Std. Dev.	Mean	*n*	Std. Dev.
Adult (18–49)	3.78	64	0.983	3.50	12	1.087
Oldish (50–64)	3.23	69	0.942	2.92	13	0.760
Young-Old (65–74)	3.04	54	0.846	3.00	23	0.953
Old (75 and over)	2.96	27	0.980	2.88	17	0.993
Total Valid	3.31	214	0.983	3.05	65	0.959
Missing		10			1	
Total		234			66	

**Table 7:** Frequency of going out.

	Bangkit		Clementi	
	*n*	%	*n*	%
>3 times per day (1)	42	18.1	15	22.7
2–3 times a day (2)	85	36.6	25	37.9
Once a day (3)	64	27.6	16	24.2
Few times every week (4)	34	14.7	9	13.6
Few times every month (5)	7	3.0	1	1.5
Total Valid	232	100.0	66	100.0
Missing	3		0	
Total	235		66	

**Table 8:** Most frequented activities in the neighbourhood (regardless of age).

	Bangkit			Clementi		
	*n*	%	% of Cases ^1^	*n*	%	% of Cases ^1^
Commuting	111	14.4	47.4	22	10.8	33.3
Strolling	79	10.2	33.8	28	13.8	42.4
Shopping	149	19.3	63.7	28	13.8	42.4
Eating	145	18.8	62.0	45	22.2	68.2
Playing	13	1.7	5.6	0	0.0	0.0
Exercising	85	11.0	36.3	26	12.8	39.4
Meeting Friends	100	12.9	42.7	31	15.3	47.0
Gardening	40	5.2	17.1	1	0.5	1.5
Visiting Community Organisations	48	6.2	20.5	21	10.3	31.8
Other	6	0.8	2.6	1	0.5	1.5
Total Answers	772	100.0	331.7	203	100.0	307.6
Total Cases	234			66		

^1^ As this was a multiple-answer question, total percentage does not equal 100%.

**Table 9:** Comparison of overall sensory appreciation assessment (Question 10 of Socio-sensory Survey, Appendix A).

	Bangkit			Clementi		
	Mean	*n*	Std. Dev.	Mean	*n*	Std. Dev.
Q10a Aesthetically appealing (H = better)	3.87	216	0.703	3.88	66	0.734
Q10b Distinguishable ambiences (H = better)	3.76	217	0.706	3.52	65	0.986
Q10c Different cultures (H = better)	3.63	209	0.852	3.52	66	0.965
Q10d Too crowded (L = better)	3.15	216	0.953	2.76	66	1.096
Q10e Clean and tidy (H = better)	3.86	217	0.851	3.42	65	1.059
Q10f Smelly (L = better)	2.44	216	0.948	2.6	65	1.101
Q10g Noisy (L = better)	2.64	217	1.076	2.5	66	1.085
Q10h Overwhelming (L = better)	2.55	211	0.972	2.63	65	1.069

Note: H = higher score; L = lower score.

**Table 10:** Spearman correlation test: correlation between sensory appreciation (Question 10 of Socio-sensory Survey, Appendix A), age and health condition.

		Bangkit		Clementi	
		Age	Health	Age	Health
Q10a Aesthetically appealing	Corr. Coef. (r)	−0.005	0.108	0.071	0.162
	Sig. (2-tailed) (p)	0.948	0.120	0.570	0.196
	N	207	209	66	65
Q10b Distinguishable ambiences	Corr. Coef. (r)	−0.067	0.108	−0.083	0.305 **
	Sig. (2-tailed) (p)	0.339	0.118	0.512	0.014
	N	207	210	65	64
Q10c Different cultures	Corr. Coef. (r)	0.030	0.052	−0.086	0.210
	Sig. (2-tailed) (p)	0.675	0.465	0.490	0.094
	N	199	202	66	65
Q10d Too crowded	Corr. Coef. (r)	−0.138 **	−0.055	−0.011	0.176
	Sig. (2-tailed) (p)	0.048	0.431	0.928	0.162
	N	206	209	66	65
Q10e Clean and tidy	Corr. Coef. (r)	0.070	0.139 **	0.157	0.065
	Sig. (2-tailed) (p)	0.319	0.044	0.210	0.612
	N	207	210	65	64
Q10f Smelly	Corr. Coef. (r)	0.018	−0.180 *	−0.027	0.056
	Sig. (2-tailed) (p)	0.792	0.009	0.832	0.661
	N	207	209	65	64
Q10g Noisy	Corr. Coef. (r)	−0.107	−0.178 *	−0.108	0.127
	Sig. (2-tailed) (p)	0.125	0.010	0.389	0.315
	N	207	210	66	65
Q10h Overwhelming	Corr. Coef. (r)	−0.009	−0.198 *	0.171	−0.051
	Sig. (2-tailed) (p)	0.898	0.004	0.174	0.692
	N	201	204	65	64

* Correlation is significant at the 0.05 level (2-tailed); ** correlation is significant at the 0.01 level (2-tailed); very weak correlations: *r* = 0.00–0.19; weak correlations: *r* = 0.20–0.39. Note: due to the small sample size in Clementi (*n* = 66), findings presented here are only indicative.

**Table 11:** Comparison of overall walking experience assessment (Question 11 of Socio-sensory Survey, Appendix A).

	Bangkit			Clementi		
	Mean	*n*	Std. Dev.	Mean	*n*	Std. Dev.
Q11a Obstacles to walk (L = better)	2.81	203	1.009	2.54	66	1.236
Q11b Slippery floors (L = better)	2.86	207	1.059	3.21	66	1.157
Q11c Hesitate to go out (L = better)	2.19	203	0.984	2.29	65	1.1
Q11d Cannot find my way (L = better)	2.08	206	0.965	2.33	66	1.114
Q11e Nature (H = better)	4.06	207	0.725	3.94	66	1.006
Q11f Avoid messy and dark places (H = better)	3.3	206	1.159	3.29	65	1.086
Q11g Prefer sheltered pathways (H = better)	4.15	204	0.805	3.73	66	0.887

Note: H = higher score; L = lower score.

**Table 12:** Spearman correlation test: correlation between walking experience (Question 11 of Socio-sensory Survey, Appendix A), age and health condition.

		Bangkit		Clementi	
		Age	Health	Age	Health
Q11a Obstacles to walk	Corr. Coef. (r)	0.037	−0.254 **	0.044	0.079
	Sig. (2-tailed) (p)	0.606	0.000	0.725	0.529
	N	193	196	66	65
Q11b Slippery floors	Corr. Coef. (r)	−0.087	−0.162 *	0.016	−0.024
	Sig. (2-tailed) (p)	0.223	0.022	0.900	0.850
	N	197	200	66	65
Q11c Hesitate to go out	Corr. Coef. (r)	0.255 **	−0.236 **	0.169	0.120
	Sig. (2-tailed) (p)	0.000	0.001	0.179	0.344
	N	193	196	65	64
Q11d Cannot find my way	Corr. Coef. (r)	0.147 *	−0.179 *	0.140	−0.029
	Sig. (2-tailed) (p)	0.040	0.011	0.262	0.817
	N	196	199	66	65
Q11e Nature	Corr. Coef. (r)	−0.191 **	0.158 *	0.055	0.115
	Sig. (2-tailed) (p)	0.007	0.026	0.661	0.360
	N	197	200	66	65
Q11f Avoid messy and dark places	Corr. Coef. (r)	−0.089	−0.162 *	0.025	−0.086
	Sig. (2-tailed) (p)	0.212	0.022	0.843	0.499
	N	197	199	65	64
Q11g Prefer sheltered pathways	Corr. Coef. (r)	−0.076	−0.038	−0.007	0.069
	Sig. (2-tailed) (p)	0.291	0.595	0.952	0.583
	N	195	197	66	65

* Correlation is significant at the 0.05 level (2-tailed); ** correlation is significant at the 0.01 level (2-tailed); very weak correlations: *r* = 0.00–0.19; weak correlations: *r* = 0.20–0.39. Note: due to the small sample size in Clementi (*n* = 66), the findings presented here are only indicative.

**Table 13:** Comparison of overall satisfaction with the neighbourhood (Question 12 of Socio-sensory Survey, Appendix A).

	Bangkit			Clementi		
	Mean	*n*	Std. Dev.	Mean	*n*	Std. Dev.
Q12a Amenities (H = better)	4.03	203	0.652	4.06	64	0.588
Q12b Different generations (H = better)	3.77	200	0.845	3.81	64	0.794
Q12c Elderly-friendly (H = better)	3.84	200	0.779	3.79	66	1.015
Q12d Happy (H = better)	4.27	203	0.702	4.24	66	0.703

Note: H = higher score; L = lower score.

**Table 14:** Spearman correlation test: correlation between overall satisfaction with the neighbourhood (Question 12 of Socio-sensory Survey, Appendix A), age and health condition.

		Bangkit		Clementi	
		Age	Health	Age	Health
Q12a Amenities	Corr. Coef. (r)	−0.009	0.151 *	−0.021	0.048
	Sig. (2-tailed) (p)	0.902	0.034	0.871	0.709
	N	194	197	64	63
Q12b Different generations	Corr. Coef. (r)	−0.108	0.002	0.154	0.120
	Sig. (2-tailed) (p)	0.138	0.982	0.223	0.348
	N	191	194	64	63
Q12c Elderly-friendly	Corr. Coef. (r)	0.040	0.262 **	0.112	0.106
	Sig. (2-tailed) (p)	0.579	0.000	0.372	0.401
	N	191	194	66	65
Q12d Happy	Corr. Coef. (r)	−0.021	0.213 **	−0.024	0.397 **
	Sig. (2-tailed) (p)	0.770	0.003	0.849	0.001
	N	194	197	66	65

* Correlation is significant at the 0.05 level (2-tailed); ** correlation is significant at the 0.01 level (2-tailed); very weak correlations: *r* = 0.00–0.19; weak correlations: *r* = 0.20–0.39. Note: Due to the small sample size in Clementi (*n* = 66), findings presented here are only indicative.

**Table 15:** Spearman correlation test: moderate correlations between different aspects of socio-perceptual assessment (Bangkit neighbourhood).

		Q10a Aesthetically Appealing	Q10g Noisy	Q10h Overwhelming	Q11a Too ManyObstacles	Q11c Hesitate to Go Out	Q12a Amenities	Q12d Happy
Q10b Distinguishable Ambiences	Corr. Coef. (r)	0.422 **						
	Sig. (2-tailed) (p)	0.000						
	N	215						
Q10f Smelly	Corr. Coef. (r)		0.447 **	0.437 **				
	Sig. (2-tailed) (p)		0.000	0.000				
	N		215	209				
Q11b Slippery Floors	Corr. Coef. (r)				0.595 **			
	Sig. (2-tailed) (p)				0.000			
	N				203			
Q11d Cannot Find My Way	Corr. Coef. (r)					0.505 **		
	Sig. (2-tailed) (p)					0.000		
	N					202		
Q12b Different Generations	Corr. Coef. (r)						0.483 **	
	Sig. (2-tailed) (p)						0.000	
	N						199	
Q12c Elderly-Friendly Design	Corr. Coef. (r)						0.438 **	0.427 **
	Sig. (2-tailed) (p)						0.000	0.000
	N						199	200
Q12d Happy	Corr. Coef. (r)						0.498 **	
	Sig. (2-tailed) (p)						0.000	
	N						202	

** Correlation is significant at the 0.01 level (2-tailed); moderate correlations: *r* = 0.40–0.59.

## Data Availability

The data presented in this study are available on request from the corresponding author. The data are not publicly available due to the agreement between the author and the Institutional Review Board of the National University of Singapore.

## Supplementary Materials

The following are available online at https://www.mdpi.com/article/10.3390/ijerph18136880/s1: Figure S1: Keywords describing the most frequently visited spaces in Bangkit neighbourhood; Figure S2: Keywords describing the most frequently visited spaces in Bangkit neighbourhood; Figure S3: Keywords describing the most liked places in Bangkit neighbourhood; Figure S4: Keywords describing the most liked places in Clementi neighbourhood; Figure S5: Keywords describing the most disliked places in Bangkit neighbourhood; Figure S6: Keywords describing the most disliked places in Clementi neighbourhood; Table S1: Time lived in the neighbourhood; Table S2: Spearman Correlation Test: Correlation between reported health condition and age increment; Table S3: Mann-Whitney U Test for reported health condition between participants with and without any sensory impairment (Bangkit sample); Table S4: Spearman Correlation Test: Correlation between reported health condition and frequency of going out; Table S5: Most frequented activities in Bangkit neighbourhood across age groups; Table S6: Most frequented activities in Clementi neighbourhood across age groups; Table S7: Most frequently visited neighbourhood places (regardless of age); Table S8: Most frequently visited places in Bangkit neighbourhood across age groups; Table S9: Most frequently visited places in Clementi neighbourhood across age groups; Table S10: Most liked neighbourhood places (regardless of age); Table S11: Most liked places in Bangkit neighbourhood across age groups; Table S12: Most liked places in Clementi neighbourhood across age groups; Table S13: Places to meet friends in the neighbourhood (regardless of age); Table S14: Places to meet friends in Bangkit neighbourhood across age groups; Table S15: Places to meet friends in Clementi neighbourhood across age groups; Table S16: Most disliked neighbourhood places (regardless of age); Table S17: Most disliked neighbourhood places in Bangkit neighbourhood across age groups; Table S18: Most disliked neighbourhood places in Clementi neighbourhood across age groups; Table S19: Overall sensory appreciation assessment in Bangkit neighbourhood (Question 10 of Socio-sensory Survey, Appendix A); Table S20: Overall sensory appreciation assessment in Clementi neighbourhood (Question 10 of Socio-sensory Survey, Appendix A); Table S21: Overall walking experience assessment in Bangkit neighbourhood (Question 11 of Socio-sensory Survey, Appendix A); Table S22: Overall walking experience assessment in Clementi neighbourhood (Question 11 of Socio-sensory Survey, Appendix A); Table S23: Overall satisfaction with Bangkit neighbourhood (Question 12 of Socio-sensory Survey, Appendix A); Table S24: Overall satisfaction with Clementi neighbourhood (Question 12 of Socio-sensory Survey, Appendix A); and Table S25: Spearman Correlations between different aspects of socio-perceptual assessment (Bangkit neighbourhood).

## Appendix A

**Table A1:** Full Socio-sensory Survey.

**General Information**
Age:
Gender: male/female
Ethnicity: Chinese/Malay/Indian/Others
How long have you lived in this neighbourhood? _________
Any sensory impairment: poor vision/poor hearing/poor smell detection/walking difficulties/cognitive difficulties/other (please specify)
Overall health condition: poor/could be better/good/very good/excellent
**Daily Routine**
1. How often do you go out of your home into your neighbourhood? (a) more than 3 times per day; (b) 2–3 times a day; (c) once a day; (d) few times every week; (e) few times every month
2. What **activities** do **you most frequently perform** within your neighbourhood (as part of your daily routine)? Choose up to 3 answers. (a) Commuting (e.g., walking to MRT station, bus stop, school, work); (b) Strolling around; (c) Shopping (e.g., going to supermarket, wet market, shopping mall); (d) Eating (e.g., in nearby foodcourt, coffee shop, restaurant); (e) Playing; (f) Jogging, exercising, cycling, etc.; (g) Meeting friends; (h) Gardening (e.g., in community garden); (i) Visiting community organisations (e.g., CC clubs, childcare, elderly care, RCs); (j) Other (please specify) _________
3. Where do you **most frequently visit** on daily basis in this neighbourhood? Name up to 2 places.
4. With regard to Question 3, please describe in any 3 keywords or phrases that reflect your feel and mood with regard to why you visit each of these places so often.
5. Where do you **like the most** in this neighbourhood? Name up to 2 places or features.
6. With regard to Question 5, please describe in 3 keywords or phrases that reflect your feel and mood with regard to why you like each of these places.
7. Where do you usually **meet your fellow neighbours or friends**? Name 1 place.
8. Where do you **dislike the most** in this neighbourhood? Name up to 2 places or features.
9. With regard to Question 8, please describe in any 3 keywords or phrases that reflect your feel and mood with regard to why you dislike these places.
**Overall Sensory Appreciation**
10. Do you agree with the following statements related to your sensory experience? (strongly disagree—1/disagree—2/neither agree nor disagree—3/somewhat agree—4/strongly agree—5)
a. This neighbourhood is overall **aesthetically appealing**.
b. This neighbourhood offers good variety of areas with **distinguishable ambients**.
c. This neighbourhood shows obvious features pertinent to **different cultures**.
d. This neighbourhood is often **too crowded**.
e. Spaces around my home are generally **clean and tidy**.
f. I often find spaces around my home **smelly**.
g. I often find spaces around my home quite **noisy**.
h. I feel **overwhelmed** and bombarded in this neighbourhood on everyday basis.
**Walking Experience**
11. Do you agree with the following statements related to your sensory experience? (strongly disagree—1/disagree—2/neither agree nor disagree—3/somewhat agree—4/strongly agree—5)
a. There are **many obstacles** (e.g., steps, dangerous curbs, gaps) **to walk** around this neighbourood.
b. Floors in this neighbourhood are **slippery** when it rains and I feel unsafe to walk.
c. I **hesitate to go out** if there is no one accompanying or helping me.
d. Sometimes, I **can’t find my way** in this neighbourhood.
e. **Nature** makes walking through this neighbourhood more enjoyable.
f. I **avoid** passing by rubbish chutes, **messy areas** and dark places.
g. I always **prefer** walking on **sheltered pathways** (covered walkways and void decks).
**Overall Satisfaction with the Neighbourhood**
12. Do you agree with the following statements related to your sensory experience? (strongly disagree—1/disagree—2/neither agree nor disagree—3/somewhat agree—4/strongly agree—5)
a. **Amenities** in this neighbourhood provide well for my daily routine needs.
b. There are plenty of opportunities for **different generations** (e.g., children and elderly) to meet.
c. This neighbourhood is overall **well-designed for the elderly** users.
d. I feel **happy** living in this neighbourhood.
