# Supplementary material for: A Study of Older Adults’ Perception of High-Density Housing Neighbourhoods in Singapore: Multi-Sensory Perspective
Source: Int J Environ Res Public Health. 2021 Jun 26;18(13):6880. doi: 10.3390/ijerph18136880 (PMC8297031; doi:10.3390/ijerph18136880)
Supplement: Supplementary file 1 [file ijerph-18-06880-s001.zip › Figures_S1-S6.pdf]

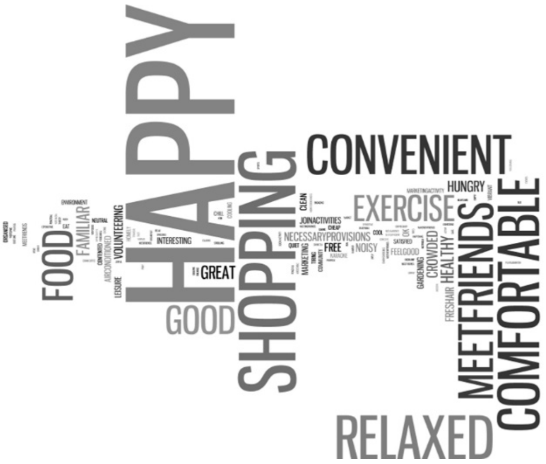

Figure S1. Keywords describing the most frequently visited spaces in Bangkit neighbourhood.

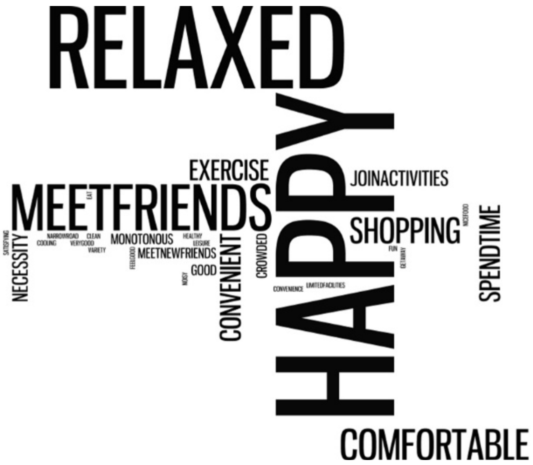

Figure S2. Keywords describing the most frequently visited spaces in Clementi neighbourhood.

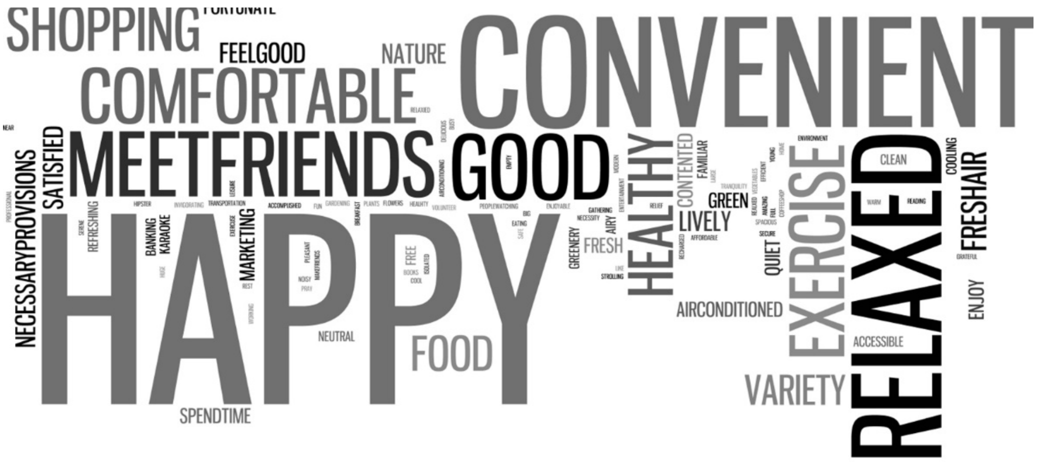

Figure S3. Keywords describing the most liked places in Bangkit neighbourhood.

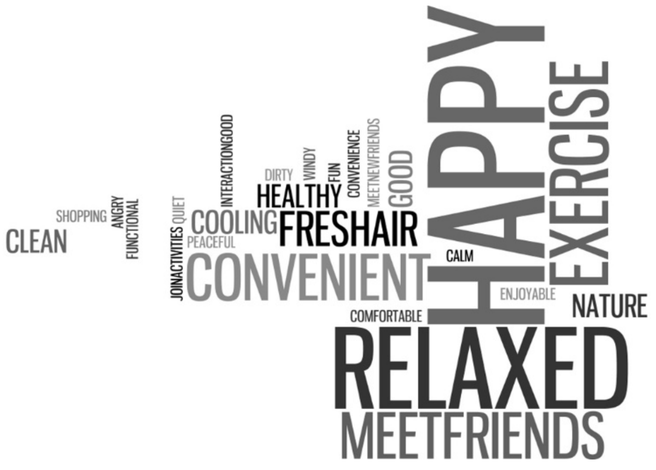

Figure S4. Keywords describing the most liked places in Clementi neighbourhood.

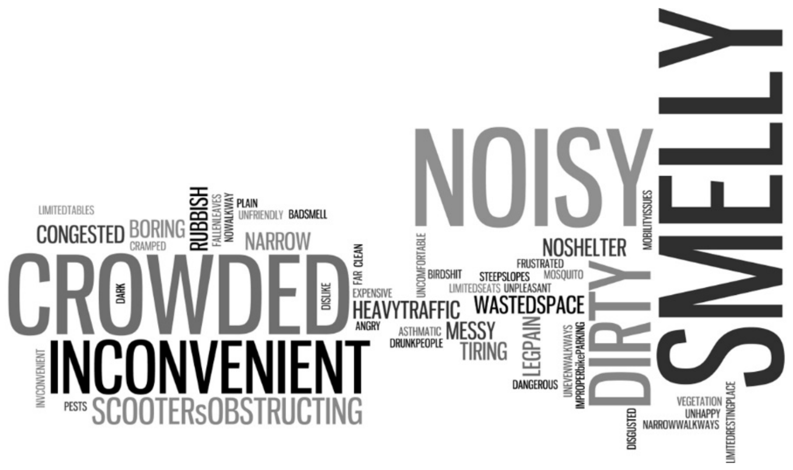

Figure S5. Keywords describing the most disliked places in Bangkit neighbourhood.

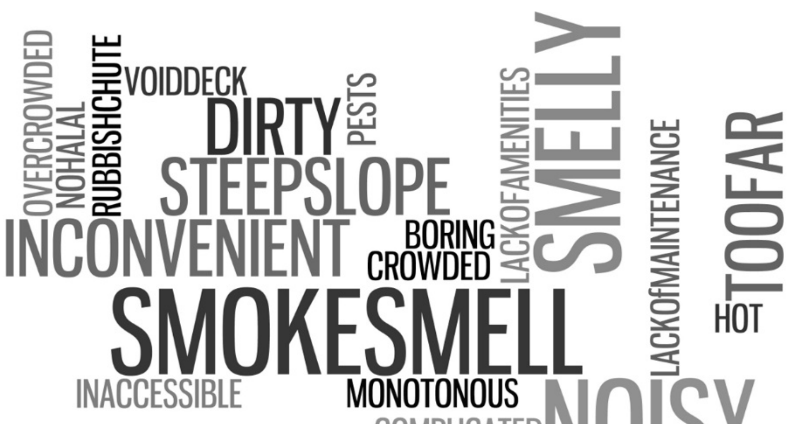

Figure S6. Keywords describing the most disliked places in Clementi neighbourhood.
