# Supplementary material for: A Study of Older Adults’ Perception of High-Density Housing Neighbourhoods in Singapore: Multi-Sensory Perspective
Source: Int J Environ Res Public Health. 2021 Jun 26;18(13):6880. doi: 10.3390/ijerph18136880 (PMC8297031; doi:10.3390/ijerph18136880)
Supplement: Supplementary file 1 [file ijerph-18-06880-s001.zip › Tables_S1-S25.pdf]

Table S1. Time lived in the neighbourhood.

	Bangkit		Clementi	
	**N**	**%**	**N**	**%**
under 1 year	0	0.0	2	3.1
1-4 years	10	4.4	5	7.8
5-19 years	42	18.6	46	71.9
over 20 years	174	77.0	11	17.2
Total Valid	226	100.0	64	100.0
Missing	9		2	
Total	235		66	

Table S2. Spearman Correlation Test: Correlation between reported health condition and age increment.

		Age	
		**Bangkit**	**Clementi**
Health condition	Corr. Coef. [r]	**-.297****	-.186
	Sig. (2-tailed) [p]	0.000	0.139
	N	214	65

** Correlation is significant at the 0.01 level (2-tailed). Weak correlation: r=0.20–0.39.

Table S3. Mann-Whitney U Test for reported health condition between participants with and without any sensory impairment (Bangkit sample).

		N	Mean Rank	Sum of Ranks
**Reported Health Condition**	No sensory imparment	169	122.82	20756.50
	Any sensory impairment	56	83.37	4668.50
	Total	225		

**Test Statistics**[a]	
	Reported Health Condition
Mann-Whitney U [U]	3072.500*
Wilcoxon W	4668.500
Z	-4.203
Asymp. Sig. (2-tailed) [p]	.000

[a] Grouping variable: Any Sensory Impairment; * Difference is significant at the 0.01 level (2-tailed).

Table S4. Spearman Correlation Test: Correlation between reported health condition and frequency of going out.

		Frequency of going out	
		**Bangkit**	**Clementi**
Health condition	Corr. Coef. [r]	**-.239****	.127
	Sig. (2-tailed) [p]	0.000	0.315
	N	223	65

* Correlation is significant at the 0.05 level (2-tailed); ** Correlation is significant at the 0.01 level (2-tailed); Weak correlation: r=0.20–0.39.

Table S5. Most frequented activities in Bangkit neighbourhood across age groups.

	18-49			50-64			65-74			74+		
	**N**	**%**	**% of Cases[1]**	**N**	**%**	**% of Cases[1]**	**N**	**%**	**% of Cases[1]**	**N**	**%**	**% of Cases[1]**
Commuting	46	20.3	70.8	36	14.8	49.3	17	9.7	30.4	7	7.1	24.1
Strolling	18	7.9	27.7	23	9.4	31.5	16	9.1	28.6	19	19.4	65.5
Shopping	41	18.1	63.1	48	19.7	65.8	35	19.9	62.5	18	18.4	62.1
Eating	51	22.5	78.5	41	16.8	56.2	32	18.2	57.1	13	13.3	44.8
Playing	7	3.1	10.8	3	1.2	4.1	3	1.7	5.4	0	0.0	0.0
Exercising	21	9.3	32.3	29	11.9	39.7	23	13.1	41.1	9	9.2	31.0
Meeting Friends	27	11.9	41.5	31	12.7	42.5	21	11.9	37.5	17	17.3	58.6
Gardening	4	1.8	6.2	16	6.6	21.9	12	6.8	21.4	8	8.2	27.6
Visiting Community Organisations	10	4.4	15.4	16	6.6	21.9	16	9.1	28.6	6	6.1	20.7
Other	2	0.9	3.1	1	0.4	1.4	1	0.6	1.8	1	1.0	3.4
Total Answers	227	100.0	349.2	244	100.0	334.2	176	100.0	314.3	98	100.0	337.9
Total Cases	65			73			56			29		

[1] As this was a multiple answer question, total percentage does not equal to 100%.

Table S6. Most frequented activities in Clementi neighbourhood across age groups.

	18-49			50-64			65-74			74+		
	**N**	**%**	**% of Cases[1]**	**N**	**%**	**% of Cases[1]**	**N**	**%**	**% of Cases[1]**	**N**	**%**	**% of Cases[1]**
Commuting	7	20.6	58.3	4	9.8	30.8	6	7.9	25.0	5	9.6	29.4
Strolling	3	8.8	25.0	6	14.6	46.2	12	15.8	50.0	7	13.5	41.2
Shopping	7	20.6	58.3	6	14.6	46.2	8	10.5	33.3	7	13.5	41.2
Eating	9	26.5	75.0	9	22.0	69.2	18	23.7	75.0	9	17.3	52.9
Playing	0	0.0	0.0	0	0.0	0.0	0	0.0	0.0	0	0.0	0.0
Exercising	6	17.6	50.0	5	12.2	38.5	6	7.9	25.0	9	17.3	52.9
Meeting Friends	1	2.9	8.3	6	14.6	46.2	15	19.7	62.5	9	17.3	52.9
Gardening	0	0.0	0.0	1	2.4	7.7	0	0.0	0.0	0	0.0	0.0
Visiting Community Organisations	1	2.9	8.3	4	9.8	30.8	11	14.5	45.8	5	9.6	29.4
Other	0	0.0	0.0	0	0.0	0.0	0	0.0	0.0	1	1.9	5.9
Total Answers	34	100.0	283.3	41	100.0	315.4	76	100.0	316.7	52	100.0	305.9
Total Cases	65			73			56			29		

[1] As this was a multiple answer question, total percentage does not equal to 100%.

Table S7. Most frequently visited neighbourhood places (regardless of age).

	Bangkit			Clementi		
	N	%	% of Cases[1]	N	%	% of Cases[1]
Shopping Spaces	101	29.9	51.0	29	31.9	45.3
Markets	68	20.1	34.3	1	1.1	1.6
Hawker and Food Centres	52	15.4	26.3	35	38.5	54.7
Community Organisations	54	16.0	27.3	18	19.8	28.1
Parks and Gardens	42	12.4	21.2	5	5.5	7.8
Fitness Spaces	5	1.5	2.5	0	0.0	0.0
Public Spaces	8	2.4	4.0	0	0.0	0.0
Others	8	2.4	4.0	3	3.3	4.7
Total Answers	338	100.0	170.7	91	100.0	142.2
No Answer	26			2		
Total Cases	198			64		

[1] As this was a multiple answer question, total percentage does not equal to 100%.

Table S8. Most frequently visited places in Bangkit neighbourhood across age groups.

	18-49			50-64			65-74			74+		
	N	%	% of Cases[1]	N	%	% of Cases[1]	N	%	% of Cases[1]	N	%	% of Cases[1]
Shopping Spaces	56	49.6	87.5	25	21.4	37.9	16	23.9	37.2	4	9.8	16.0
Markets	15	13.3	23.4	31	26.5	47.0	12	17.9	27.9	10	24.4	40.0
Hawker and Food Centres	15	13.3	23.4	19	16.2	28.8	13	19.4	30.2	5	12.2	20.0
Community Organisations	19	16.8	29.7	19	16.2	28.8	8	11.9	18.6	8	19.5	32.0
Parks and Gardens	5	4.4	7.8	16	13.7	24.2	13	19.4	30.2	8	19.5	32.0
Fitness Spaces	0	0.0	0.0	2	1.7	3.0	2	3.0	4.7	1	2.4	4.0
Public Spaces	0	0.0	0.0	2	1.7	3.0	1	1.5	2.3	5	12.2	20.0
Others	3	2.7	4.7	3	2.6	4.5	2	3.0	4.7	0	0.0	0.0
Total Answers	113	100.0	176.6	117	100.0	177.3	67	100.0	155.8	41	100.0	164.0
No Answer	1			7			13			5		
Total Cases	64			66			43			25		

[1] As this was a multiple answer question, total percentage does not equal to 100%.

Table S9. Most frequently visited places in Clementi neighbourhood across age groups.

	18-49			50-64			65-74			74+		
	N	%	% of Cases[1]	N	%	% of Cases[1]	N	%	% of Cases[1]	N	%	% of Cases[1]
Shopping Spaces	11	61.1	91.7	6	30.0	46.2	8	25.8	34.8	4	18.2	25.0
Markets	0	0.0	0.0	0	0.0	0.0	0	0.0	0.0	1	4.5	6.3
Hawker and Food Centres	4	22.2	33.3	5	25.0	38.5	16	51.6	69.6	10	45.5	62.5
Community Organisations	0	0.0	0.0	6	30.0	46.2	6	19.4	26.1	6	27.3	37.5
Parks and Gardens	1	5.6	8.3	2	10.0	15.4	1	3.2	4.3	1	4.5	6.3
Fitness Spaces	0	0.0	0.0	0	0.0	0.0	0	0.0	0.0	0	0.0	0.0
Public Spaces	0	0.0	0.0	0	0.0	0.0	0	0.0	0.0	0	0.0	0.0
Others	2	11.1	16.7	1	5.0	7.7	0	0.0	0.0	0	0.0	0.0
Total Answers	18	100.0	150.0	20	100.0	153.8	31	100.0	134.8	22	100.0	137.5
No Answer	0			0			1			1		
Total Cases	12			13			23			16		

[1] As this was a multiple answer question, total percentage does not equal to 100%.

Table S10. Most liked neighbourhood places (regardless of age).

	Bangkit			Clementi		
	**N**	**%**	**% of Cases[1]**	**N**	**%**	**% of Cases[1]**
Shopping Spaces	70	25.7	42.4	10	16.9	20.4
Markets	35	12.9	21.2	0	0.0	0.0
Hawker and Food Centres	24	8.8	14.5	17	28.8	34.7
Community Organisations	51	18.8	30.9	14	23.7	28.6
Parks and Gardens	67	24.6	40.6	18	30.5	36.7
Fitness Spaces	2	0.7	1.2	0	0.0	0.0
Public Spaces	9	3.3	5.5	0	0.0	0.0
Others	14	5.1	8.5	0	0.0	0.0
Total Answers	272	100.0	164.8	59	100.0	120.4
No Answer	59			17		
Total Cases	165			49		

[1] As this was a multiple answer question, total percentage does not equal to 100%.

Table S11. Most liked places in Bangkit neighbourhood across age groups.

	18-49			50-64			65-74			74+		
	**N**	**%**	**% of Cases[1]**	**N**	**%**	**% of Cases[1]**	**N**	**%**	**% of Cases[1]**	**N**	**%**	**% of Cases[1]**
Shopping Spaces	32	33.0	56.1	23	25.0	41.8	12	21.8	33.3	3	10.7	17.6
Markets	7	7.2	12.3	15	16.3	27.3	9	16.4	25.0	4	14.3	23.5
Hawker and Food Centres	7	7.2	12.3	11	12.0	20.0	2	3.6	5.6	4	14.3	23.5
Community Organisations	15	15.5	26.3	14	15.2	25.5	16	29.1	44.4	6	21.4	35.3
Parks and Gardens	26	26.8	45.6	24	26.1	43.6	12	21.8	33.3	5	17.9	29.4
Fitness Spaces	0	0.0	0.0	1	1.1	1.8	0	0.0	0.0	1	3.6	5.9
Public Spaces	1	1.0	1.8	2	2.2	3.6	2	3.6	5.6	4	14.3	23.5
Others	9	9.3	15.8	2	2.2	3.6	2	3.6	5.6	1	3.6	5.9
Total Answers	97	100.0	170.2	92	100.0	167.3	55	100.0	152.8	28	100.0	164.7
No Answer	8			18			20			13		
Total Cases	57			55			36			17		

[1] As this was a multiple answer question, total percentage does not equal to 100%.

Table S12. Most liked places in Clementi neighbourhood across age groups.

	18-49			50-64			65-74			74+		
	**N**	**%**	**% of Cases[1]**	**N**	**%**	**% of Cases[1]**	**N**	**%**	**% of Cases[1]**	**N**	**%**	**% of Cases[1]**
Shopping Spaces	2	15.4	22.2	3	18.8	25.0	3	16.7	17.6	2	16.7	18.2
Markets	0	0.0	0.0	0	0.0	0.0	0	0.0	0.0	0	0.0	0.0
Hawker and Food Centres	3	23.1	33.3	3	18.8	25.0	5	27.8	29.4	6	50.0	54.5
Community Organisations	2	15.4	22.2	4	25.0	33.3	4	22.2	23.5	4	33.3	36.4
Parks and Gardens	6	46.2	66.7	6	37.5	50.0	6	33.3	35.3	0	0.0	0.0
Fitness Spaces	0	0.0	0.0	0	0.0	0.0	0	0.0	0.0	0	0.0	0.0
Public Spaces	0	0.0	0.0	0	0.0	0.0	0	0.0	0.0	0	0.0	0.0
Others	0	0.0	0.0	0	0.0	0.0	0	0.0	0.0	0	0.0	0.0
Total Answers	13	100.0	144.4	16	100.0	133.3	18	100.0	105.9	12	100.0	109.1
No Answer	3			1			7			6		
Total Cases	9			12			17			11		

[1] As this was a multiple answer question, total percentage does not equal to 100%.

Table S13. Places to meet friends in the neighbourhood (regardless of age).

	Bangkit		Clementi	
	N	%	N	%
Shopping Spaces	27	15.4	3	5.9
Markets	7	4.0	1	2.0
Hawker and Food Centres	74	42.3	31	60.8
Community Organisations	19	10.9	5	9.8
Parks and Gardens	13	7.4	2	3.9
Fitness Spaces	1	0.6	0	0.0
Public Spaces	7	4.0	0	0.0
Others	27	15.4	9	17.6
Total Answers	175	100.0	51	100.0
No Answer	49		15	
Total Cases	27	15.4	3	5.9

Table S14. Places to meet friends in Bangkit neighbourhood across age groups.

	18-49		50-64		65-74		74+	
	N	%	N	%	N	%	N	%
Shopping Spaces	20	32.8	5	8.1	1	2.9	1	5.9
Markets	1	1.6	5	8.1	1	2.9	0	0.0
Hawker and Food Centres	25	41.0	25	40.3	18	51.4	6	35.3
Community Organisations	6	9.8	10	16.1	2	5.7	1	5.9
Parks and Gardens	2	3.3	9	14.5	1	2.9	1	5.9
Fitness Spaces	0	0.0	0	0.0	1	2.9	0	0.0
Public Spaces	0	0.0	1	1.6	3	8.6	3	17.6
Others	7	11.5	7	11.3	8	22.9	5	29.4
Total Answers	61	100.0	62	100.0	35	100.0	17	100.0
No Answer	4		11		21		13	

Table S15. Places to meet friends in Clementi neighbourhood across age groups.

	18-49		50-64		65-74		74+	
	N	%	N	%	N	%	N	%
Shopping Spaces	2	20.0	1	9.1	0	0.0	0	0.0
Markets	1	10.0	0	0.0	0	0.0	0	0.0
Hawker and Food Centres	3	30.0	6	54.5	15	78.9	7	63.6
Community Organisations	0	0.0	1	9.1	1	5.3	3	27.3
Parks and Gardens	1	10.0	1	9.1	0	0.0	0	0.0
Fitness Spaces	0	0.0	0	0.0	0	0.0	0	0.0
Public Spaces	0	0.0	0	0.0	0	0.0	0	0.0
Others	3	30.0	2	18.2	3	15.8	1	9.1
Total Answers	10	100.0	11	100.0	19	100.0	11	100.0
No Answer	2		2		5		6	

Table S16. Most disliked neighbourhood places (regardless of age).

	Bangkit			Clementi		
	N	%	% of Cases[1]	N	%	% of Cases[1]
Shopping Spaces	4	4.8	5.9	1	3.8	4.5
Markets	6	7.1	8.8	0	0.0	0.0
Hawker and Food Centres	7	8.3	10.3	6	23.1	27.3
Community Organisations	2	2.4	2.9	1	3.8	4.5
Parks and Gardens	1	1.2	1.5	2	7.7	9.1
Fitness Spaces	2	2.4	2.9	0	0.0	0.0
Public Spaces	5	6.0	7.4	0	0.0	0.0
Others[2]	57	67.9	83.8	16	61.5	72.7
Total Answers	84	100.0	123.5	26	100.0	118.2
No Answer	156			44		
Total Cases	68			22		

[1] As this was a multiple answer question, total percentage does not equal to 100%.
[2] These spaces typically refer to messy or dark areas close to rubbish collection points.

.

Table S17. Most disliked neighbourhood places in Bangkit neighbourhood across age groups.

	18-49			50-64			65-74			74+		
	N	%	% of Cases[1]	N	%	% of Cases[1]	N	%	% of Cases[1]	N	%	% of Cases[1]
Shopping Spaces	2	6.5	8.0	1	3.2	4.0	0	0.0	0.0	1	25.0	33.3
Markets	1	3.2	4.0	0	0.0	0.0	5	27.8	33.3	0	0.0	0.0
Hawker and Food Centres	2	6.5	8.0	2	6.5	8.0	2	11.1	13.3	1	25.0	33.3
Community Organisations	2	6.5	8.0	0	0.0	0.0	0	0.0	0.0	0	0.0	0.0
Parks and Gardens	1	3.2	4.0	0	0.0	0.0	0	0.0	0.0	0	0.0	0.0
Fitness Spaces	0	0.0	0.0	0	0.0	0.0	1	5.6	6.7	1	25.0	33.3
Public Spaces	1	3.2	4.0	1	3.2	4.0	3	16.7	20.0	0	0.0	0.0
Others[2]	22	71.0	88.0	27	87.1	108.0	7	38.9	46.7	1	25.0	33.3
Total Answers	31	100.0	124.0	31	100.0	124.0	18	100.0	120.0	4	100.0	133.3
No Answer	40			48			41			27		
Total Cases	25			25			15			3		

[1] As this was a multiple answer question, total percentage does not equal to 100%.
[2] These spaces typically refer to messy or dark areas close to rubbish collection points.

Table S18. Most disliked neighbourhood places in Clementi neighbourhood across age groups.

	18-49			50-64			65-74			74+		
	N	%	% of Cases[1]	N	%	% of Cases[1]	N	%	% of Cases[1]	N	%	% of Cases[1]
Shopping Spaces	1	12.5	14.3	0	0.0	0.0	0	0.0	0.0	0	0.0	0.0
Markets	0	0.0	0.0	0	0.0	0.0	0	0.0	0.0	0	0.0	0.0
Hawker and Food Centres	2	25.0	28.6	3	33.3	42.9	1	14.3	16.7	0	0.0	0.0
Community Organisations	0	0.0	0.0	0	0.0	0.0	0	0.0	0.0	1	50.0	50.0
Parks and Gardens	0	0.0	0.0	1	11.1	14.3	1	14.3	16.7	0	0.0	0.0
Fitness Spaces	0	0.0	0.0	0	0.0	0.0	0	0.0	0.0	0	0.0	0.0
Public Spaces	0	0.0	0.0	0	0.0	0.0	0	0.0	0.0	0	0.0	0.0
Others[2]	5	62.5	71.4	5	55.6	71.4	5	71.4	83.3	1	50.0	50.0
Total Answers	8	100.0	114.3	9	100.0	128.6	7	100.0	116.7	2	100.0	100.0
No Answer	5			6			18			15		
Total Cases	7			7			6			2		

[1] As this was a multiple answer question, total percentage does not equal to 100%.
[2] These spaces typically refer to messy or dark areas close to rubbish collection points.

Table S19. Overall sensory appreciation assessment in Bangkit neighbourhood (Question 10 of Socio-sensory Survey, Appendix A).

	Strongly Disagree		Somewhat Disagree		Neither Agree nor Disagree		Somewhat Agree		Strongly Agree		Total	
	N	%	N	%	N	%	N	%	N	%	N	%
Q10a Aesthetically appealing	2	0.93	7	3.24	36	16.67	143	66.20	28	12.96	216	100
Q10b Distinguishable ambients	1	0.46	9	4.15	53	24.42	132	60.83	22	10.14	217	100
Q10c Different cultures	5	2.39	14	6.70	57	27.27	111	53.11	22	10.53	209	100
Q10d Too crowded	6	2.78	52	24.07	76	35.19	68	31.48	14	6.48	216	100
Q10e Clean & tidy	4	1.84	15	6.91	27	12.44	133	61.29	38	17.51	217	100
Q10f Smelly	31	13.14	94	39.83	62	26.27	24	10.17	25	10.59	235	100
Q10g Noisy	31	14.29	77	35.48	56	25.81	45	20.74	8	3.69	217	100
Q10h Overwhelming	29	13.74	78	36.97	66	31.28	35	16.59	3	1.42	211	100

Table S20. Overall sensory appreciation assessment in Clementi neighbourhood (Question 10 of Socio-sensory Survey, Appendix A).

	Strongly Disagree		Somewhat Disagree		Neither Agree nor Disagree		Somewhat Agree		Strongly Agree		Total	
	N	%	N	%	N	%	N	%	N	%	N	%
Q10a Aesthetically appealing	1	1.52	2	3.03	10	15.15	44	66.67	9	13.64	66	100
Q10b Distinguishable ambients	4	6.15	4	6.15	18	27.69	32	49.23	7	10.77	65	100
Q10c Different cultures	3	4.55	7	10.61	15	22.73	35	53.03	6	9.09	66	100
Q10d Too crowded	3	4.55	34	51.52	11	16.67	12	18.18	6	9.09	66	100
Q10e Clean & tidy	2	3.08	15	23.08	9	13.85	32	49.23	7	10.77	65	100
Q10f Smelly	9	13.85	27	41.54	13	20.00	13	20.00	3	4.62	65	100
Q10g Noisy	8	12.12	34	51.52	12	18.18	7	10.61	5	7.58	66	100
Q10h Overwhelming	5	7.69	33	50.77	13	20.00	9	13.85	5	7.69	65	100

Table S21. Overall walking experience assessment in Bangkit neighbourhood (Question 11 of Socio-sensory Survey, Appendix A).

	Strongly Disagree		Somewhat Disagree		Neither Agree nor Disagree		Somewhat Agree		Strongly Agree		Total	
	N	%	N	%	N	%	N	%	N	%	N	%
Q11a Obstacles to walk	13	6.40	81	39.90	47	23.15	56	27.59	6	2.96	203	100
Q11b Slippery floors	16	7.73	76	36.71	44	21.26	63	30.43	8	3.86	207	100
Q11c Hesitate to go out	48	23.65	98	48.28	31	15.27	22	10.84	4	1.97	203	100
Q11d Cannot find my way	60	29.13	95	46.12	29	14.08	19	9.22	3	1.46	206	100
Q11e Nature	1	0.48	6	2.90	24	11.59	124	59.90	52	25.12	207	100
Q11f Avoid messy & dark places	7	3.40	60	29.13	38	18.45	66	32.04	35	16.99	206	100
Q11g Prefer sheltered pathways	10	2.45	23	5.64	98	24.02	73	17.89	204	50.00	408	100

Table S22. Overall walking experience assessment in Clementi neighbourhood (Question 11 of Socio-sensory Survey, Appendix A).

	Strongly Disagree		Somewhat Disagree		Neither Agree nor Disagree		Somewhat Agree		Strongly Agree		Total	
	N	%	N	%	N	%	N	%	N	%	N	%
Q11a Obstacles to walk	9	13.64	31	46.97	9	13.64	9	13.64	8	12.12	66	100
Q11b Slippery floors	2	3.03	24	36.36	6	9.09	26	39.39	8	12.12	66	100
Q11c Hesitate to go out	14	21.54	32	49.23	9	13.85	6	9.23	4	6.15	65	100
Q11d Cannot find my way	13	19.70	33	50.00	10	15.15	5	7.58	5	7.58	66	100
Q11e Nature	3	4.55	4	6.06	5	7.58	36	54.55	18	27.27	66	100
Q11f Avoid messy & dark places	4	6.15	13	20.00	14	21.54	28	43.08	6	9.23	65	100
Q11g Prefer sheltered pathways	2	3.03	3	4.55	16	24.24	35	53.03	10	15.15	66	100

Table S23. Overall satisfaction with Bangkit neighbourhood (Question 12 of Socio-sensory Survey, Appendix A).

	Strongly Disagree		Somewhat Disagree		Neither Agree nor Disagree		Somewhat Agree		Strongly Agree		Total	
	N	%	N	%	N	%	N	%	N	%	N	%
Q12a Amenities	0	0.00	5	2.46	25	12.32	132	65.02	41	20.20	203	100
Q12b Different Generations	4	2.00	13	6.50	37	18.50	118	59.00	28	14.00	200	100
Q12c Elderly-friendly	3	1.50	9	4.50	34	17.00	125	62.50	29	14.50	200	100
Q12d Happy	1	0.33	2	0.66	18	5.94	79	26.07	203	67.00	303	100

Table S24. Overall satisfaction with Clementi neighbourhood (Question 12 of Socio-sensory Survey, Appendix A).

	Strongly Disagree		Somewhat Disagree		Neither Agree nor Disagree		Somewhat Agree		Strongly Agree		Total	
	N	%	N	%	N	%	N	%	N	%	N	%
Q12a Amenities	0	0.00	2	3.13	3	4.69	48	75.00	11	17.19	64	100
Q12b Different Generations	0	0.00	7	10.94	6	9.38	43	67.19	8	12.50	64	100
Q12c Elderly-friendly	3	4.55	7	10.61	3	4.55	41	62.12	12	18.18	66	100
Q12d Happy	0	0.00	2	3.03	4	6.06	36	54.55	24	36.36	66	100

Table S25. Spearman Correlations between different aspects of socio-perceptual assessment (Bangkit neighbourhood).

		Q10a	Q10b	Q10c	Q10d	Q10e	Q10f	Q10g	Q10h	Q11a	Q11b	Q11c	Q11d	Q11e	Q11f	Q11g	Q12a	Q12b	Q12c	Q12d
Q10a Aesthetically Appealing	Corr. Coef.		.422**	.227**	-0.047	.193**	-0.080	-.177**	-.154*	-.164*	-0.091	-.219**	-0.102	.148*	-0.079	.153*	.368**	0.136	.252**	.370**
	Sig. (2-tailed)		0.000	0.001	0.491	0.004	0.245	0.009	0.025	0.021	0.196	0.002	0.148	0.036	0.265	0.031	0.000	0.059	0.000	0.000
	N		215	208	214	215	214	215	210	198	202	198	201	202	201	199	197	194	194	197
Q10b Distinguishable Ambients	Corr. Coef.	.422**		.292**	-0.024	0.103	-0.052	-0.002	-0.049	-0.009	0.053	-0.103	-0.042	.242**	0.057	.198**	.334**	.336**	.323**	.263**
	Sig. (2-tailed)	0.000		0.000	0.731	0.132	0.451	0.979	0.482	0.897	0.448	0.148	0.553	0.000	0.417	0.005	0.000	0.000	0.000	0.000
	N	215		208	215	216	215	216	211	200	204	200	203	204	203	201	199	196	196	199
Q10c Different Cultures	Corr. Coef.	.227**	.292**		0.054	0.129	-0.058	-0.112	0.032	-0.102	-0.073	-0.033	-0.004	.185**	-0.021	.155*	.165*	.208**	.265**	.258**
	Sig. (2-tailed)	0.001	0.000		0.441	0.062	0.405	0.106	0.646	0.158	0.309	0.652	0.953	0.009	0.769	0.030	0.022	0.004	0.000	0.000
	N	208	208		208	209	208	208	204	192	196	193	195	196	195	195	191	190	190	191
Q10d Too Crowded	Corr. Coef.	-0.047	-0.024	0.054		-0.020	0.103	.185**	.357**	.159*	0.102	0.047	0.093	-0.047	0.017	0.090	-0.028	0.122	-0.050	-0.036
	Sig. (2-tailed)	0.491	0.731	0.441		0.768	0.131	0.006	0.000	0.025	0.148	0.513	0.190	0.507	0.811	0.205	0.699	0.090	0.493	0.613
	N	214	215	208		215	214	215	209	199	202	198	201	202	201	199	197	194	194	197
Q10e Clean and Tidy	Corr. Coef.	.193**	0.103	0.129	-0.020		-.348**	-.355**	-0.076	-.143*	-.159*	-.179*	-.230**	.179*	-0.123	0.070	.250**	.153*	.307**	.195**
	Sig. (2-tailed)	0.004	0.132	0.062	0.768		0.000	0.000	0.275	0.044	0.024	0.011	0.001	0.011	0.081	0.324	0.000	0.033	0.000	0.006
	N	215	216	209	215		215	216	210	199	203	199	202	203	202	200	198	195	195	198
Q10f Smelly	Corr. Coef.	-0.080	-0.052	-0.058	0.103	-.348**		.447**	.437**	.256**	.276**	.338**	.355**	-0.115	.242**	-0.029	-.178*	-0.141	-0.084	-.159*
	Sig. (2-tailed)	0.245	0.451	0.405	0.131	0.000		0.000	0.000	0.000	0.000	0.000	0.000	0.103	0.001	0.686	0.012	0.050	0.241	0.026
	N	214	215	208	214	215		215	209	198	202	198	201	202	202	200	198	195	195	198
Q10g Noisy	Corr. Coef.	-.177**	-0.002	-0.112	.185**	-.355**	.447**		.341**	.303**	.297**	.235**	.197**	-0.099	.280**	0.022	-.191**	-0.046	-0.086	-.176*
	Sig. (2-tailed)	0.009	0.979	0.106	0.006	0.000	0.000		0.000	0.000	0.000	0.001	0.005	0.158	0.000	0.753	0.007	0.519	0.234	0.013
	N	215	216	208	215	216	215		210	199	203	199	202	203	202	200	198	195	195	198
Q10h Sensory Overwhelmed	Corr. Coef.	-.154*	-0.049	0.032	.357**	-0.076	.437**	.341**		.271**	.299**	.282**	.252**	-0.131	.283**	0.112	-0.108	-0.089	-.202**	-.260**
	Sig. (2-tailed)	0.025	0.482	0.646	0.000	0.275	0.000	0.000		0.000	0.000	0.000	0.000	0.065	0.000	0.116	0.134	0.220	0.005	0.000
	N	210	211	204	209	210	209	210		195	199	196	198	199	198	197	195	193	193	195
Q11a Too Many Obstacles	Corr. Coef.	-.164*	-0.009	-0.102	.159*	-.143*	.256**	.303**	.271**		.595**	.379**	.367**	0.101	.295**	0.051	-.160*	-0.032	-.184*	-.235**
	Sig. (2-tailed)	0.021	0.897	0.158	0.025	0.044	0.000	0.000	0.000		0.000	0.000	0.000	0.151	0.000	0.473	0.024	0.654	0.010	0.001
	N	198	200	192	199	199	198	199	195		203	198	201	202	201	199	197	194	194	197
Q11b Slippery Floors	Corr. Coef.	-0.091	0.053	-0.073	0.102	-.159*	.276**	.297**	.299**	.595**		.353**	.340**	0.068	.348**	0.091	-.181*	-0.039	-0.110	-0.120
	Sig. (2-tailed)	0.196	0.448	0.309	0.148	0.024	0.000	0.000	0.000	0.000		0.000	0.000	0.333	0.000	0.199	0.010	0.591	0.123	0.091
	N	202	204	196	202	203	202	203	199	203		202	205	206	205	203	199	197	197	200
Q11c Hesitate to Go Out	Corr. Coef.	-.219**	-0.103	-0.033	0.047	-.179*	.338**	.235**	.282**	.379**	.353**		.505**	-0.092	.149*	0.060	-.189**	-.182*	0.059	-0.137
	Sig. (2-tailed)	0.002	0.148	0.652	0.513	0.011	0.000	0.001	0.000	0.000	0.000		0.000	0.195	0.034	0.399	0.008	0.011	0.416	0.056
	N	198	200	193	198	199	198	199	196	198	202		202	202	201	200	195	193	193	195
Q11d Cannot Find My Way	Corr. Coef.	-0.102	-0.042	-0.004	0.093	-.230**	.355**	.197**	.252**	.367**	.340**	.505**		-0.118	.149*	-0.096	-.261**	-.228**	-0.094	-.208**
	Sig. (2-tailed)	0.148	0.553	0.953	0.190	0.001	0.000	0.005	0.000	0.000	0.000	0.000		0.090	0.033	0.171	0.000	0.001	0.188	0.003
	N	201	203	195	201	202	201	202	198	201	205	202		206	205	203	199	196	196	199
Q11e Nature	Corr. Coef.	.148*	.242**	.185**	-0.047	.179*	-0.115	-0.099	-0.131	0.101	0.068	-0.092	-0.118		0.110	.319**	.302**	.304**	.247**	.331**
	Sig. (2-tailed)	0.036	0.000	0.009	0.507	0.011	0.103	0.158	0.065	0.151	0.333	0.195	0.090		0.115	0.000	0.000	0.000	0.000	0.000
	N	202	204	196	202	203	202	203	199	202	206	202	206		206	204	200	197	197	200
Q11f Messy and Dark Places	Corr. Coef.	-0.079	0.057	-0.021	0.017	-0.123	.242**	.280**	.283**	.295**	.348**	.149*	.149*	0.110		.316**	-0.050	0.062	-0.123	-0.058
	Sig. (2-tailed)	0.265	0.417	0.769	0.811	0.081	0.001	0.000	0.000	0.000	0.000	0.034	0.033	0.115		0.000	0.484	0.388	0.085	0.416
	N	201	203	195	201	202	202	202	198	201	205	201	205	206		204	200	197	197	200
Q11g Sheltered Pathways	Corr. Coef.	.153*	.198**	.155*	0.090	0.070	-0.029	0.022	0.112	0.051	0.091	0.060	-0.096	.319**	.316**		.241**	.300**	.232**	.186**
	Sig. (2-tailed)	0.031	0.005	0.030	0.205	0.324	0.686	0.753	0.116	0.473	0.199	0.399	0.171	0.000	0.000		0.001	0.000	0.001	0.009
	N	199	201	195	199	200	200	200	197	199	203	200	203	204	204		198	197	197	198
Q12a Amenities	Corr. Coef.	.368**	.334**	.165*	-0.028	.250**	-.178*	-.191**	-0.108	-.160*	-.181*	-.189**	-.261**	.302**	-0.050	.241**		.483**	.438**	.498**
	Sig. (2-tailed)	0.000	0.000	0.022	0.699	0.000	0.012	0.007	0.134	0.024	0.010	0.008	0.000	0.000	0.484	0.001		0.000	0.000	0.000
	N	197	199	191	197	198	198	198	195	197	199	195	199	200	200	198		199	199	202
Q12b Different Generations	Corr. Coef.	0.136	.336**	.208**	0.122	.153*	-0.141	-0.046	-0.089	-0.032	-0.039	-.182*	-.228**	.304**	0.062	.300**	.483**		.386**	.345**
	Sig. (2-tailed)	0.059	0.000	0.004	0.090	0.033	0.050	0.519	0.220	0.654	0.591	0.011	0.001	0.000	0.388	0.000	0.000		0.000	0.000
	N	194	196	190	194	195	195	195	193	194	197	193	196	197	197	197	199		199	200
Q12c Elderly-friendly Design	Corr. Coef.	.252**	.323**	.265**	-0.050	.307**	-0.084	-0.086	-.202**	-.184*	-0.110	0.059	-0.094	.247**	-0.123	.232**	.438**	.386**		.427**
	Sig. (2-tailed)	0.000	0.000	0.000	0.493	0.000	0.241	0.234	0.005	0.010	0.123	0.416	0.188	0.000	0.085	0.001	0.000	0.000		0.000
	N	194	196	190	194	195	195	195	193	194	197	193	196	197	197	197	199	199		200
Q12d Happy	Corr. Coef.	.370**	.263**	.258**	-0.036	.195**	-.159*	-.176*	-.260**	-.235**	-0.120	-0.137	-.208**	.331**	-0.058	.186**	.498**	.345**	.427**	
	Sig. (2-tailed)	0.000	0.000	0.000	0.613	0.006	0.026	0.013	0.000	0.001	0.091	0.056	0.003	0.000	0.416	0.009	0.000	0.000	0.000	
	N	197	199	191	197	198	198	198	195	197	200	195	199	200	200	198	202	200	200	

**. Correlation is significant at the 0.01 level (2-tailed).

*. Correlation is significant at the 0.05 level (2-tailed).

Very Weak (r=0.00-0.19)	Weak (r=0.20-0.39)	Moderate (r=0.40-0.59)	Strong (r=0.60-0.79)	Very Strong (r=0.80-1.00)
